# Sclerozoan and fouling sabellid worms (Annelida: Sabellidae) from Mexico with the establishment of two new species

**DOI:** 10.3897/BDJ.8.e57471

**Published:** 2020-10-08

**Authors:** María Ana Ana Tovar-Hernández, María Elena García-Garza, Jesús Angel de León-González

**Affiliations:** 1 Universidad Autónoma de Nuevo León, Facultad de Ciencias Biológicas, Laboratorio de Biosistemática, Nuevo Léon, Mexico Universidad Autónoma de Nuevo León, Facultad de Ciencias Biológicas, Laboratorio de Biosistemática Nuevo Léon Mexico; 2 Universidad Autónoma de Nuevo León, Facultad de Ciencias Biológicas, Laboratorio de Biosistemática, Nuevo León, Mexico Universidad Autónoma de Nuevo León, Facultad de Ciencias Biológicas, Laboratorio de Biosistemática Nuevo León Mexico

**Keywords:** Bioclaustration, *
Anamobaea
*, *
Notaulax
*, eastern Tropical Pacific, Gulf of California, Veracruz, Acapulco, fan worms.

## Abstract

**Background:**

The sabellid genera *Anamobaea* Krøyer, 1856 and *Notaulax* Tauber, 1879 are two of the most attractive polychaete worms in coral reefs. *Anamobaea* is represented by two Caribbean species and *Notaulax* with 24 species from around the world, six of them previously known to tropical America. During examination of fouling biota and sclerozoans from Mexico, *Anamobaea orstedii* Krøyer, 1856 was found in coral reefs from the southern Gulf of Mexico and three species of *Notaulax* were identified to the Mexican Pacific, two of them being new species to science.

**New information:**

*Anamobaea orstedii* Krøyer, 1856 is first reported as sclerozoan of dead coral from the southern Gulf of Mexico. An amendment to the generic diagnosis of *Anamobaea* is provided, based on the presence of a higher number of skeletal cells than previously recorded; height, shape and exposition of the anterior peristomial ring; the non-fusion of dorsal collar margins to faecal groove; shape of collar chaetiger and abdominal chaetae and distribution and shape of radiolar ocelli. *Notaulax californica* (Treadwell, 1906) is reported as fouling in buoys and docks from the Gulf of California. Two new species of *Notaulax* are described: the former was found in hull and dock fouling from La Paz (Gulf of California) and the second one as sclerozoan of oysters from a dock fouling in Acapulco (south Mexican Pacific). In addition, reproductive features are described for the first time for *A. orstedii* which is a simultaneous hermaphrodite with female and males gametes found within the same segments of abdominal region. Oocytes develop synchronously and sperm morphology (spherical nucleus and rounded acrosome, four spherical mitochondria and a long free flagellum) suggest an adaptation to broadcast spawning and external fertilisation. Species of *Notaulax* here examined were gonochoric, with gametes distributed in abdominal segments.

## Introduction

The sabellid genus *Anamobaea* Krøyer, 1856 (Krøyer 1856), is one of the most attractive polychaete worms from Caribbean coral reefs due to their large, colourful radiolar crowns. *Anamobaea* is represented by two species: the type species *A. orstedii* Krøyer, 1856 (Krøyer 1856), described from the West Indies and *A. phyllisae* Tovar-Hernández and Salazar-Vallejo, 2006 (Tovar-Hernández and Salazar-Vallejo 2006), from the British Virgin Islands. Both species have been reported as sclerozoan in dead coral masses from several Caribbean localities (San Martín et al. 1994; Humman and Deloach 2001; Tovar-Hernández and Salazar-Vallejo 2006). Both are commonly known as “split crown feather duster worm”, perhaps due its appearance in two halves when its crown is fully open or “ghost feather duster” because, when some kind of threat or disturbance is around, worms elicit a rapid defensive retraction into their protective tube hiding their crowns.

*Anamobaea* is closely related to the genera *Hypsicomus* Grube, 1870 (Grube 1870) and *Notaulax* Tauber, 1879 (Tauber 1879). These three genera have been nested in a well-defined clade defined by the presence of simple radiolar ocelli and paleate chaetae in the anterior row of abdominal neurochaetal fascicles Fitzhugh (1989). Based on a recent analysis of phylogenomics, *Hypsicomus* sp. and *Anamobaea orstedii* were placed as the most apomorphic group within Sabellidae Tilic et al. (2020).

*Hypsicomus* is monotypic, with *H. stichophthalmos* (Grube, 1863) (Grube 1863) from the Mediterranean Sea, whereas *Notaulax* is currently composed by 24 species after Nishi et al. (2017), Tovar-Hernández et al. (2017) and Tovar-Hernández et al. (2020). Eight species of *Notaulax* are known to bioclaustrate into coral masses, one of those was described as sclerozoan from the living coral *Montipora nodosa* (Dana, 1846) (Dana 1846) from Indonesia (Tovar-Hernández et al. 2020); one was found associated with a fossil reef; other species have been found fouling in marinas and ports (Tovar-Hernández et al. 2017) and for others, there is no information on the substrates from which they were collected (Nishi et al. 2017).

Seven species of *Notaulax* have been previously reported to Tropical America and Mexico: *N. bahamensis* Perkins, 1984 (Perkins 1984), *N. californica* (Treadwell, 1906) (Treadwell 1906), *N. circumspiciens* (Ehlers, 1887) (Ehlers 1887), *N. midoculi* (Hoagland, 1919) (Hoagland 1919), *N. nudicollis* (Krøyer, 1856) (Krøyer 1856), *N. occidentalis* (Baird, 1865) (Baird 1865) and *N. paucoculata* Perkins, 1984 (Perkins 1984; Tovar-Hernández and Salazar-Vallejo 2006; Tovar-Hernández and Salazar-Silva 2008; Tovar-Hernández 2009; Miranda Salinas et al. 2016; Yáñez-Rivera et al. 2020).

In this study, *A. orstedii* is first recorded to the southern Gulf of Mexico, revealing that some diagnostic features were misinterpreted or omitted in previous contributions, dealing with additions to the generic description, as well as information about reproductive mode and gamete distribution and shape. In addition, three species of *Notaulax* are recorded from western Mexico, two of them are new species to science of sclerozoan and fouling sabellids, respectively.

## Taxon treatments

### Anamobaea

Krøyer, 1856

A40F74A2-BB3E-5FDA-A62C-B7ADBFD6C333

Anamobaea *Anamobaea orstedii*
Anamobaea *Anamöbæa Ørstedii*
Anamobaea .— Fitzhugh 1989: 74; Capa et al. 2019: 191–192.
Anamobaea **Diagnosis (amended)**
Anamobaea *sensu*[Bibr B5994585][Bibr B5994710][Bibr B5994603]

#### Taxon discussion

*Anamobaea* was placed in synonymy with *Hypsicomus* by Augener (1925) and defended by Hartman (1959). However, Perkins (1984) recognised *Anamobaea*, stating that *A. orstedii* has dorsal and ventral basal flanges (as *Notaulax*), not present in *Hypsicomus*.

*Anamobaea*, *Hypsicomus* and *Notaulax* form part of a well-defined clade, being *Anamobaea* plesiomorphic to *Notaulax* and *Hypsicomus*, the latter two genera being sister taxa, based on the common occurrence of radiolar flanges (Fitzhugh 1989). In a posterior analysis, the three genera were also nested together, but *Hypsicomus* resulted in being plesiomorphic to *Anamobaea* and *Notaulax* (Nogueira et al. 2010).

*Anamobaea* is represented by two species worldwide that have been only reported in dead coral masses (bioclaustration). Eight species of *Notaulax* are known to bioclaustrate into coral masses as well (Nishi et al. 2017, Tovar-Hernández et al. 2020). It is unknown where the substrate of *Hypsicomus stichophthalmos* Grube, 1863 (Grube 1863) was found.

Major differences amongst *Anamobaea*, *Hypsicomus* and *Notaulax* are the following: *Hypsicomus* has two pairs of accessory, auriculate lamellae, absent in *Anamobaea*. *Anamobaea* and *Hypsicomus* have chaetae of collar arranged in a small bunch, whereas in *Notaulax*, collar chaetal arrangement may be longitudinal, oblique, L-shaped, J-shaped or C-shaped. Members of *Anamobaea* do not present radiolar flanges, but these structures are common in *Hypsicomus* and *Notaulax*, amongst other differences (Table 1).

The present definition primarily follows Fitzhugh (1989) and Capa et al. (2019), except for the following: the specimens here examined have 12–16 vacuolated cells in cross section at the base (four or more vacuolated cells in Fitzhugh (1989) and Capa et al. (2019)); the anterior peristomial ring is high, with rounded margin (low, of even height in in Fitzhugh 1989 and Capa et al. 2019); dorsal collar margins are not fused to faecal groove (fused to faecal groove in Capa et al. 2019); chaetae form a collar arranged in a small bundle (arrangement not described in Fitzhugh 1989 or Capa et al. 2019); mucros of paleate chaetae in abdominal chaetigers are dentate (mucros not described in Fitzhugh 1989 and Capa et al. 2019), those mucros from anterior abdominal segments are short (as long as paleae width) while those from posterior abdominal segments are long (longer than three times the width of paleae). Additional features related to radiolar ocelli were based on Bok et al. (2016).

### Anamobaea orstedii

Krøyer, 1856

09F3CDE6-9866-577E-AA96-2EA77D085505

Anamobaea orstedii *Anamöbæa Ørstedii*
Anamobaea orstedi. — Humman and Deloach 2001: 155; San Martín et al. 1994: 556, fig. 1A–I; Tovar-Hernández and Salazar-Vallejo 2006: 27–30, figs. 1–2.

#### Materials

- **Type status:** Other material. **Occurrence:** catalogNumber: UANL 8133; recordedBy: Luis Fernando Carrera-Parra, Isabel Cristina Molina-Acevedo, Tulio Fabio Villalobos-Guerrero, Sergio I. Salazar-Vallejo; individualCount: 8; sex: Hermaphrodite; lifeStage: Adult; reproductiveCondition: Ripe; **Taxon:** phylum: Annelida; class: Polychaeta; order: Sabellida Levinsen, 1883; family: Sabellidae Latreille, 1825; genus: Anamobaea Krøyer, 1856; specificEpithet: *orstedii* Krøyer, 1856; **Location:** higherGeography: Atlantic Ocean; continent: America; waterBody: Gulf of Mexico; islandGroup: Sistema Arrecifal Veracruzano; country: México; countryCode: MX; stateProvince: Veracruz de Ignacio de la Llave; municipality: Veracruz; locality: Isla Verde; maximumDepthInMeters: 1 m; decimalLatitude: 19.201191; decimalLongitude: -96.065768; **Identification:** identifiedBy: María Ana Tovar-Hernández; **Event:** samplingProtocol: By hand; eventDate: 27March 2015; year: 2015; month: 03; day: 27; habitat: Dead coral; **Record Level:** language: Spanish; institutionID: Universidad Autónoma de Nuevo León; collectionID: Colección Poliquetológica, Universidad Autónoma de Nuevo León; institutionCode: UANL; collectionCode: UANL, NL INV 0002-05-09

#### Description

Figures 1–5

*Body shape and size*. Specimens fairly large and plump on thorax, flattened dorso-ventrally in thorax (Fig. 2B), pyriform abdomen in transversal section (Fig. 3K). Body length 10–42 mm (n = 5, all lacking posterior abdomen), width 1.5–4 mm.

*Radiolar crown.* Length 6–17 mm with 15–20 pairs of radioles. Base of radiolar crown (basal lamina) smooth, long, as long as the length of eight thoracic segments in largest specimens. Erect, prominent dorsal and ventral flanges (Fig. 2A–F and Fig. 3A–B). Dorsal flanges forming a broad and deep canal from the base of dorsalmost radioles until base of posterior peristomial ring collar (Fig. 2C and Fig. 3B). Ventral flanges with an anterior digitiform lobe overlapped (Fig. 2A) and exposing the base of parallel lamellae. Radioles united by a palmate membrane as long as two times the length of base of crown or basal lamina (Fig. 2F). Radiolar skeleton composed of 12–16 cells in cross section at the base (Fig. 3F), then decreasing in number towards mid-radiolar length (Fig. 3J). Longest pinnules located at three quarters of the radiolar crown length. Radiolar tips filiform, occupying the space of 5–8 pinnules, without flanges (Fig. 4H). Ocelli in both sides of the outside of radioles (Fig. 3G–I). Dorsal most radioles with ocelli in groups of 15–22: basal ocelli distributed in two irregular rows (Fig. 3H), distal ocelli forms only one row (Fig. 3I). Ventral radioles with 5–10 ocelli in a single row. Dorsal lips long, erect, triangular (Fig. 3E) with mid-rib (radiolar appendage). Dorsal pinnular appendages present, short. Dorsal lips longer than palmate membrane, reaching the basalmost radiolar ocelli. Ventral lips small, folded and joining radiolar lobes near origin of first ventral radiole. Ventral radiolar appendages absent.

*Peristomium*. Anterior peristomial ring high, as long as the length of three thoracic segments, with rounded margin (Fig. 4A–B, crown removed). Posterior peristomial ring collar: dorsal margins shallow, not fused to faecal groove (Fig. 3B); ventral margins forming two nearly triangular lappets (Fig. 2A and D), not overlapped (whereas relaxed or contracted). Parallel lamellae and ventral sacs present (Fig. 3D). Ventral sacs rounded, inflated and exposed outside ventral basal flanges of crown, in some specimens full of sand (Fig. 3A and D). Dorsal margin of anterior peristomial ring exposed above collar margin (posterior peristomial ring), triangular, divided by faecal groove (Fig. 3B).

*Thorax.* Chaetigers very numerous (20–54 chaetigers). Ventral shield of collar rectangular, longer and broader than following shields (Fig. 2A). Collar chaetae arranged in a bunch (Fig. 4A) of short, spine-like with symmetrical hoods (Fig. 4D). Ventral shields of chaetigers 2–8 rectangular, swollen (Fig. 2A). Superior notochaetae spine-like, short, 3–4 per group (Fig. 5A). Inferior notochaetae paleate, symmetrical (Fig. 4E) or asymmetrical (Fig. 5B), arranged in two transverse rows, each row with 3–4 chaetae (Fig. 5A). Tori not contacting ventrals shields (Fig. 2A). Uncini avicular, with minute, equal-size teeth covering half of the crest (Fig. 5E), breast well developed, handle as long as two times the length of crest (Fig. 4I). Companion chaetae with symmetrical tips as teardrop-shaped membranes (Fig. 4I, Fig. 5D), shaft not longer than uncini. Posterior segments with brown blood vessels dorsally, forming rectangular, sinuous nets seen in both alive and fixed material (Fig. 3C).

*Abdomen*. Number of chaetigers not fully determined (incomplete specimens, the longest with 124 segments). Segments with brown blood vessels dorsally, forming rectangular, sinuous nets (Fig. 3C). Neurochaetal fascicles in two transverse rows. Anterior abdominal segments with anterior and posterior rows of paleate chaetae with dentate mucros (Fig. 4G and Fig. 5C), mucros short (as long as paleae width) being fragile and broken in many paleae (Fig. 5C) and modified, elongate, narrowly-hooded chaetae (Fig. 4K). Posterior abdominal segments with paleae with long mucros (more than three times the width of paleae) (Fig. 4G) and modified, elongate, narrowly-hooded chaetae. Uncini avicular, with 30–37 rows of equal-sized teeth covering 3/4 of the crest length (Fig. 5F), breast well developed (Fig. 4J), handles as long as crest. Pygidium not examined (specimens lacking the last segments of abdomen, near pygidium).

*Variation.* One large specimen (44 thoracic chaetigers, 3.5 mm width) with a regenerating radiolar crown: it is short, measuring only 6 mm in length with 18 pairs of radioles, all unequal in length. This regenerating crown has a palmate membrane well developed and dorsal radioles with rows of 8–14 ocelli, whereas ventral radioles have 5–6 ocelli.

**Figure 1.**
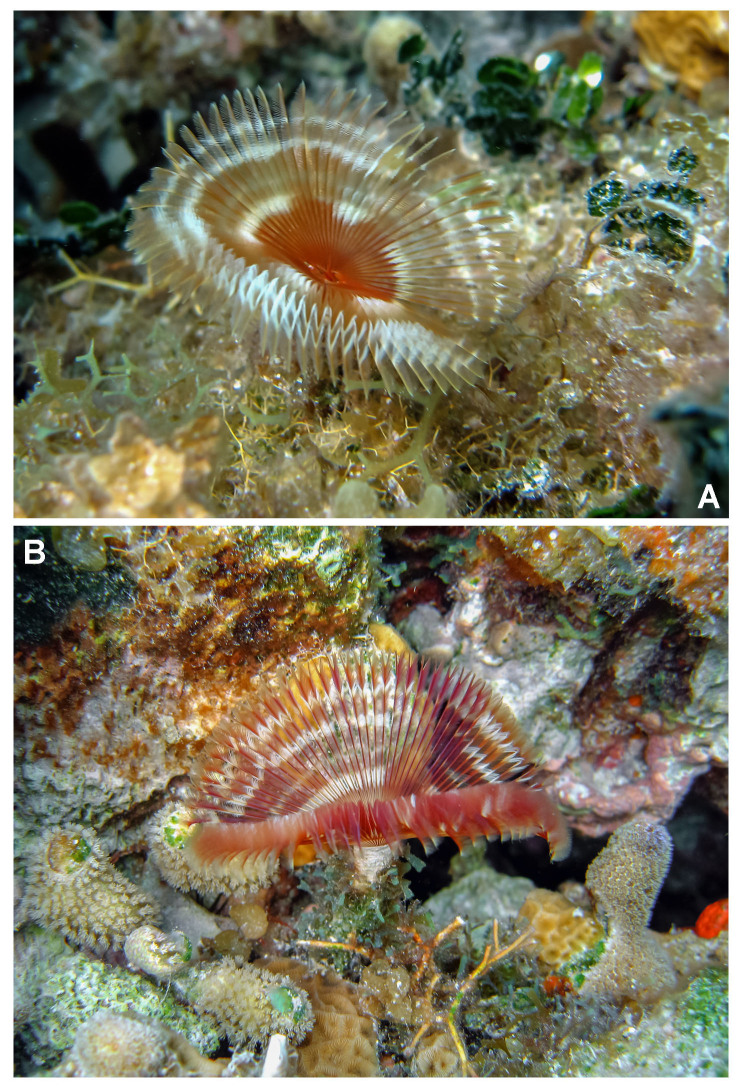
*Anamobaea orstedii* in coral reefs from southern Gulf of Mexico.

**Figure 2.**
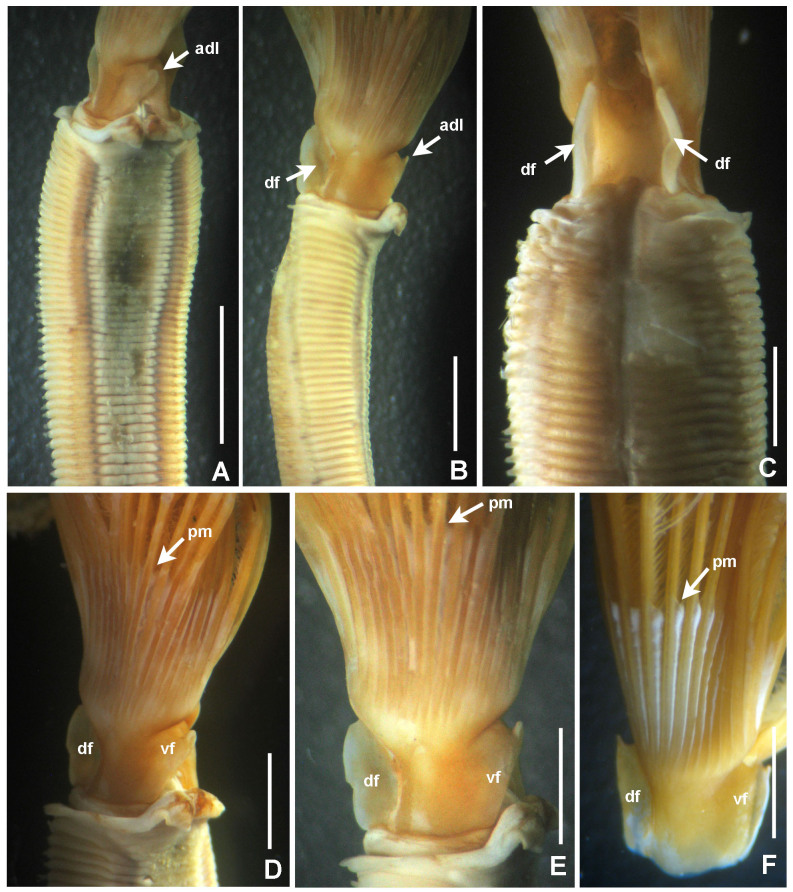
Selected features of *Anamobaea orstedii*. A–C) Thoracic segments and base of radiolar crown, D–F) base of radiolar crown and radioles. A) Ventral view, B, D–F) lateral views, C) dorsal view. Abbreviations: adl) anterior digitiform lobe, df) dorsal flange, pm) palmate membrane, vf) ventral flange. Scale bars: A) 4 mm, B–C) 2 mm, D–F) 1.5 mm. A–C) UANL 8133.

*Colour in live specimens*. Radiolar crown with red and white bands *in situ* (Fig. 1). Under light microscopy, base of radiolar crown translucent. Basal half of radiolar crown red with a translucent palmate membrane and red radiolar ocelli. Distal half of radiolar crown with alternate white and red bands. Ventral lappets of posterior peristomial ring collar and lateral margin of anterior peristomial ring red. Body yellow with ventral shields and lateral margins of the body whitish. Anterior thoracic segments with narrow, red, transversal lines between each torus.

*Colour in preserved specimens.* Palmate membrane white. Basal half of crown with radioles brown to orange containing rows of brown ocelli. Distal half of crown with whitish radioles. Radiolar tips white. Ventral lappets with large, orange spots each. Whitish ventral sacs. Segments from mid-thorax with two brown, sinuous blood vessels dorsally forming a nearly-rectangular shape. Some abdominal segments with brown, sinuous blood vessels forming a rectangular shape dorsally.

*Tubes.* Tubes embedded in dead coral seem like wooden trunks with a strong consistency, composed of an external wide bark-like layer and several thin, golden internal layers.

#### Diagnosis

Base of the radiolar crown smooth (basal lamina) with prominent, erect, dorsal and ventral flanges. Collar chaetae arranged in a bunch. Ocelli in both sides of the outer margin of radioles. Dorsal-most radioles with ocelli in groups of 15–22 (basal ocelli distributed in two rows, distal ocelli forms only one row). Ventral radioles with 5–10 ocelli in a single row. Simultaneous hermaphrodite.

#### Biology

Ripe simultaneous hermaphrodites were found with female and male gametes within the coelom of abdominal segments and also attached to the internal tube layer (Fig. 4K–L). Oocytes are equal in size and spermatozoa with spherical nucleus and rounded cap-like acrosome.

#### Taxon discussion

According to Fitzhugh (1989), members of *Anamobaea*, *Hypsicomus* and *Notaulax*, have a low anterior margin of the anterior peristomial ring, of even height all around. However, in the description provided here for *Anamobaea*, this feature is interpreted as high (as long as three thoracic segments), with distal margin rounded once crowns were removed to properly examine the peristomium (Fig. 4B–C). In addition, Perkins (1984)[figs. 25E–F and 35E–F], illustrates anterior peristomial rings of *Notaulax bahamensis* Perkins, 1984 (Perkins 1984) and *N. nudicollis* (Krøyer, 1856) (Krøyer 1856) similar to that described here for *Anamobaea*. Consequently, a re-examination of this feature is needed in *Hypsicomus*, as well as in other members of *Notaulax*.

Tovar-Hernández and Salazar-Vallejo (2006)reported a radiolar skeleton composed of 6–7 cells in cross-section and thorax with 32–55 chaetigers. In the present study, a small variation was found: 12–16 cells at the base of radioles and then decreasing in number towards medium radiole length. Specimens here reported have 20–54 thoracic chaetigers. Ventral lips were incorrectly shown by Tovar-Hernández and Salazar-Vallejo (2006) in their figure 1B (as vl). These structures correspond to ventral sacs, not ventral lips. The ventral lips are small, folded and joining radiolar lobes near the origin of first ventral radiole.

In addition, it should be noted that the spelling of the species name *orstedii* has previously been incorrect in a number of publications (misspelled as “*orstedi*” instead the original “*orstedii*”) in Fitzhugh (1989)[p. 74], San Martín et al. (1994) [p. 556], Tovar-Hernández and Salazar-Vallejo (2006) [p. 27], Dean (2012) [p. 65] and Capa et al. (2019) [p. 191].

Detailed information about eye and ocelli types found in *A. orstedii* was provided by Bok et al. (2016): eye type S (scattered ocelli on both sides of each radiole) and ocelli type 4 (four-cell forming the ocellus).

Reproductive information is absent for *Anamobaea*. In this study, the presence of simultaneous hermaphroditism is confirmed in *A. orstedii.* Brooding within the tube of the radiolar crown was not observed. Broadcast spawning is supported by sperm morphology (spherical heads).

Most of the sabellid species present a thorax consisting typically of eight chaetigers (Fitzhugh 1989), but there is a remarkable exception in *Anamobaea*, where a long thorax consisting of 20–54 chaetigerous segments in *Anamobaea orstedii* (this study) or 74 in *A. phyllisae* has been reported, being the highest number of thoracic segments reported nowadays in Sabellidae (Tovar-Hernández and Salazar-Vallejo 2006). Other exceptions are represented by members of *Perkinsiana*, which may have 19 thoracic segments as in *P. anodina* or 24 in *P. longa* (Capa 2007).

Perhaps the presence of a long thorax in *Anamobaea* and some species of *Perkinsiana* is related to their mode of life in corals. *Perkinsiana anodina* was described as sclerozoan of dead coral from Western Australia, where there were large granite boulders with small colonies of live and dead corals on them (Capa 2007). It was also found in Tiger Island (Indonesia) surrounded by tissue of a mushroom coral where their tubes form straight protuberances on the scleractinian coral surface (Tovar-Hernández et al. 2020). As corals provided refuge to worms in addition to that provided by their own tubes, it is probable that worms present a high growth and longevity as demonstrated in other infaunal, tube worms of corals such as the serpulid *Spirobranchus corniculatus* (Grube, 1862) (*Grube 1862*) with an estimated longevity of 15–20 years or the sabellariid *Idanthyrsus* sp., with eight years of longevity (Nishi and Nishihira 1999, also see their fig. 1 where a boring *Notaulax* was sketched). The tube ultrastructure of *A. orstedii* has a regular plywood microstructure which is lamellar in cross-section (Vinn et al. 2018), whereas the tube wall of *Perkinsiana anodina* is dense, non porous, with sparsely spaced three different sets of fibres (Tovar-Hernández et al. 2020). This indicates that inhabiting with coral has had no effect on the tube microstructure of the species, possibly because *P. anodina* did not communicate with the host coral through its tube wall.

### Notaulax

Tauber, 1879

B1629B72-0025-5888-82AA-3BC6A711CA25

Notaulax *Notaulax rectangulata*Levinsen 1883
Notaulax Tauber, 1879 (Tauber 1879): 136.
Notaulax .— Perkins 1984: 327, 329; Fitzhugh 1989: 75; Tovar-Hernández et al. 2017: 21; Capa et al. 2019: 197–198; Tovar-Hernández et al. 2020: 27–28.

#### Diagnosis

Radioles in semi-circular radiolar lobes, each radiole with at least four rows of vacuolated cells. Radiolar crown with elongate basal lobes; palmate membrane, radiolar flanges and dorsal and ventral basal flanges present. Numerous ocelli arranged in longitudinal rows on lateral sides of radioles. Dorsal lips with radiolar appendages, pinnular appendages absent; ventral radiolar appendages absent. Ventral lips and parallel lamellae present, ventral sacs inside radiolar crown. Anterior peristomial ring low, of even height, or high and rounded. Posterior peristomial ring collar with narrow mid-dorsal gap, dorsal margins laterally fused to the faecal groove, ventrally entire or with mid-ventral incision and short ventral lappets. Peristomial vascular loops absent. Peristomial eyespots absent. Thorax and abdomen with variable number of segments. Glandular ridge on chaetiger 2 absent. Ventral shields present. Interramal eyespots may be present. Collar chaetae spine-like, arranged in distally oblique longitudinal rows, diagonal, J or C-inverted shaped; superior thoracic notochaetae short spine-like, inferior thoracic notochaetae paleate. Thoracic uncini avicular, with several rows of minute and similar in size teeth above main fang, developed breast and medium-sized handle; neuropodial companion chaetae with strongly asymmetrical hood, stouter on one margin and thin, elongate tip. Abdominal uncini similar to the thoracic ones. Anterior abdomen with a superior group of elongate, narrowly-hooded chaetae and an inferior group of paleate chaetae with mucros. Posterior abdomen with modified, elongate, narrowly-hooded chaetae and paleate chaetae (spherical or oval) with long mucros. Pygidial eyespots may be present. Anal cirrus absent (after Tovar-Hernández et al. 2020).

#### Taxon discussion

Perkins (1984) revised the genus, described new species, provided several synonyms and proposed new combinations of species within *Notaulax*. Since then, *Notaulax* sp. 1, *Notaulax* sp. 2 and *Notaulax* sp. 3 have been reported from Lizard Island by Capa and Murray (2015). *Notaulax yamasui* Nishi et al. 2017 was established to Japan. *Sabella tilosaula* Schmarda, 1861 (Schmarda 1861) was transferred to *Notaulax* and a new species was described from Puerto Deseado (Argentina) as *Notaulax salazari* by Tovar-Hernández et al. (2017). Lastly, the genus was amended by Tovar-Hernández et al. (2020) who also recognised three species from Indonesia within *Notaulax*: *Sabella pyrrhogaster* Grube, 1878 (Grube 1878), Sabella (Potamilla) tenuitorques Grube, 1878 (Grube 1878) and *Notaulax montiporicola* Tovar-Hernández and ten Hove, 2020 (Tovar-Hernández et al. 2020). Despite these efforts, the genus is waiting for a worldwide revision as supported below.

The generic diagnosis by Fitzhugh (1989) stated that chaetae from the anterior abdomen are elongate, narrowly-hooded, whereas chaetae from the posterior abdomen are modified, elongate, narrowly-hooded chaetae. Then, the diagnosis provided by Capa et al. (2019) refers to the abdominal chaetae as needle-like without any differentiation between anterior and posterior abdomen. Needle-like chaetae *sensu Fitzhugh (1989)* [Fig. 24A] are straight capillaries present in some serpulids as *Protula* Risso, 1826 which are different from those present in *Notaulax* (slightly curved, thin, long and narrowly hooded). It is hard to obtain complete specimens since these are embedded within strong tubes in dead or living coral, rocks or as fouling. However, below we designated the holotype of *N. nigrofouling* sp. n., from the Gulf of California based in a complete specimen. We confirm that these abdominal chaetae are, in fact, narrowly hooded which usually become progressively longer towards the pygidium (where these are referred as modified, elongate, narrowly hooded).

As Tovar-Hernández et al. (2020) pointed out, chaetae from the anterior half of the abdomen are different from those in the posterior half of the abdomen in *Notaulax*. Chaetae from the anterior abdomen are paleate, nearly rounded or spherical with short mucros (as long as palea width), whereas chaetae from the posterior abdomen are spherical or oval, but with a long mucro, longer than three times the width of paleae. In *Notaulax montiporicola*, the presence of two types of mucros in the abdominal paleate chaetae is remarkable: with candle flame-shaped mucros in anterior abdominal segments, sail-shaped mucros posteriorly. This combination of abdominal chaetae has never been detected before in *Notaulax*, but it might be an important feature to distinguish genera. In *Anamobaea*, chaetae from the posterior abdomen are paleate, but with long mucros in contrast with those from the anterior abdominal segments in which the mucro is short (as long as palea width).

Chaetae from the collar have been called spine-like *sensu* Perkins (1984) or Fitzhugh (1989). Moore and Bush (1904) realised that, in *Notaulax lyra* (*as Hypsicomus*), the collar chaetae are arranged in a double series along each line, but those spine-like chaetae are slightly different to each other. Spine-like chaetae of the dorsal-most series are stouter, nearly straight, terminated by an elongated conical hood or sheath more or less inflated at the base and usually bent or wavy in the slender distal half (see Moore and Bush 1904: plate XI, figure 8). Those of the ventral-most series are more slender, sharply curved at the end and provided on the convex side with a short but broad obliquely-striated wing (see Moore and Bush 1904: plate XI, figure 7). This differentiation could be useful to genera level since it has been unnoticed in most contributions.

Perkins (1984) recommended as characters of specific importance the arrangement and position of radiolar ocelli, the shape of collar, the number of thoracic chaetigers, the cross sectional structure of the radioles and pronounced differences in the chaetae and uncini. However, the number of thoracic chaetigers is variable due ontogeny. Assessing sections of radioles to count the number of skeletal cells and lengths of flanges is not practical at the specific level (it is informative and useful to genera level in some genera). First, these are impacted by anaesthetics and fixation methods. Second, the number of cells and flanged extension lengths will depend of where the sections are done (basally, where the basal lamina ends or in an intermediate zone where the palmate membrane is). Third, if sections are hand-made using scissors, razor blades or scalpel, often this procedure makes thick sections near to 1 mm width, in contrast with those assessed using a microtome were sections are in microns. Consequently, the number of cells can vary in some order of magnitude depending on the method to sectioning radioles and different interpretations by researchers.

Variability of some other features were emphasised by Perkins (1984). He remarked that the collar of some species change during development from a 4-lobed structure on juveniles to a 1 or 2 lobed structure on adults, which cannot be corroborated in this study. In many specimens of *N. nigroincrustata* sp. n., reviewed here (six types and 14 additional specimens), the ventral margin of the collar is always entire, but it has two variations: even in height (as lateral margins of collar) or slightly higher than lateral margins of collar; and the lateral collar margins can be even in height or asymmetrical V-shaped.

As stated by Perkins (1984), in juveniles, the position and organisation of ocelli are not diagnostic. It was also corroborated in *N. californica* where juveniles have five ocelli or up to 16 ocelli in adults (see below). In addition, the length of the basal lamina (or radiolar lobes) is also variable as demonstrated in this study by *N. nigroincrustata* sp. n.

**Figure 3.**
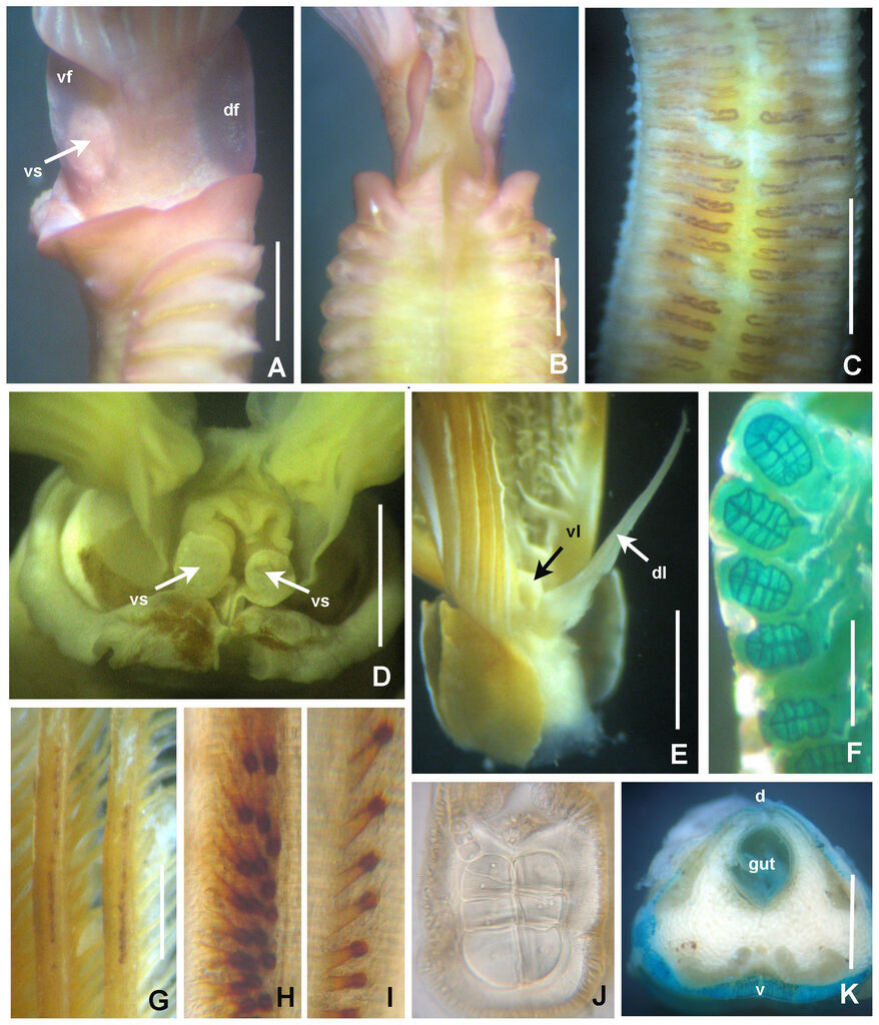
Selected features of *Anamobaea orstedii*. A–B) Base of radiolar crown, C) thoracic segments, dorsal view, D) radiolar crown internal structures, E) dorsal and ventral lip, F) base of radioles showing skeletal cells, cross section, G) radiolar ocelli, H) detail of basal ocelli, I) detail of distal ocelli, J) cross section of radiole at mid-radiole length, K) abdomen, transversal section. Abbreviations: d) dorsum, df) dorsal flange, dl) dorsal lip, v) ventrum, vf) ventral flange, vl) ventral lip, vs) ventral sacs. Scale bars: A–B) 0.5 mm, C) 1.75 mm, D) 1 mm, E) 0.75 mm, F–G) 0.25 mm, H–J) not scaled, K) 0.4 mm. A–K) UANL 8133.

**Figure 4.**
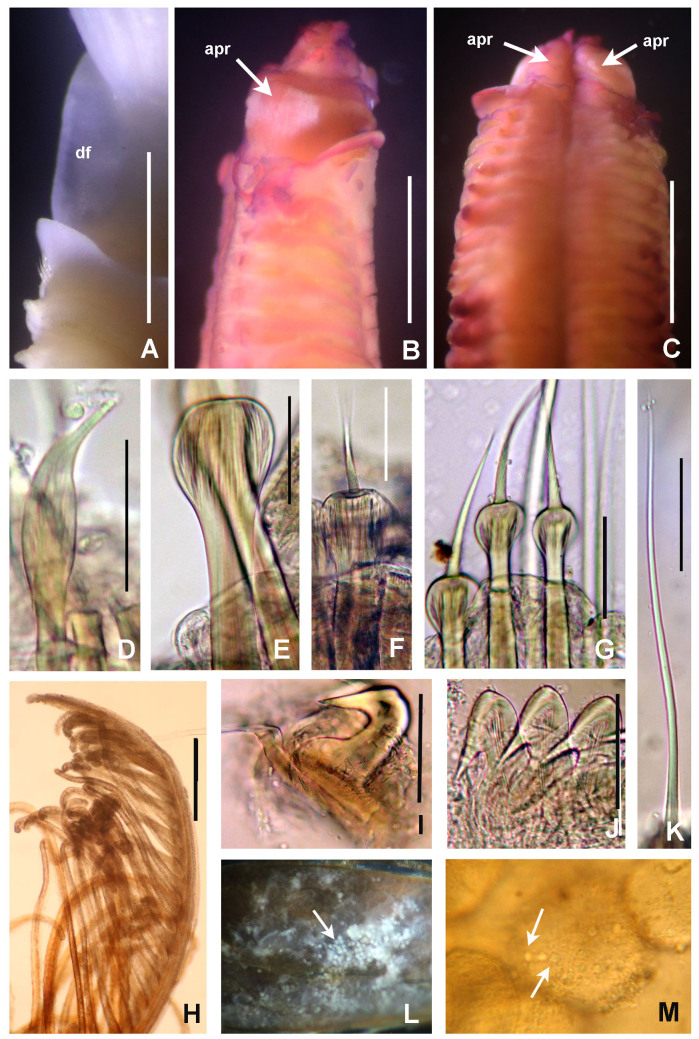
Selected features of *Anamobaea orstedii.* A) Collar chaetiger and dorsal flange, B) anterior peristomial ring, crown removed, dorso-lateral view, C) same, dorsal view, D) spine-like chaeta from collar, E) thoracic paleate chaeta, F) paleate chaeta from anterior half of abdomen, G) paleate chaetae from posterior abdominal segments, H) radiolar tip, I) thoracic uncinus and companion chaetae, J) abdominal uncini, K) elongate, modified narrowly-hooded chaetae from abdomen, L) oocytes and sperm attached to the internal layer of tube, M) oocytes and spermatozoa indicated with arrows. Abbreviations: apr) anterior peristomial ring, df) dorsal flange. Scale bars: A) 1 mm, B–C) 0.8 mm, D–G, K) 30 μm, H) 0.6 mm, I–J) 24 μm, L) not scaled, M) 1000x magnification. A–M) UANL 8133.

**Figure 5.**
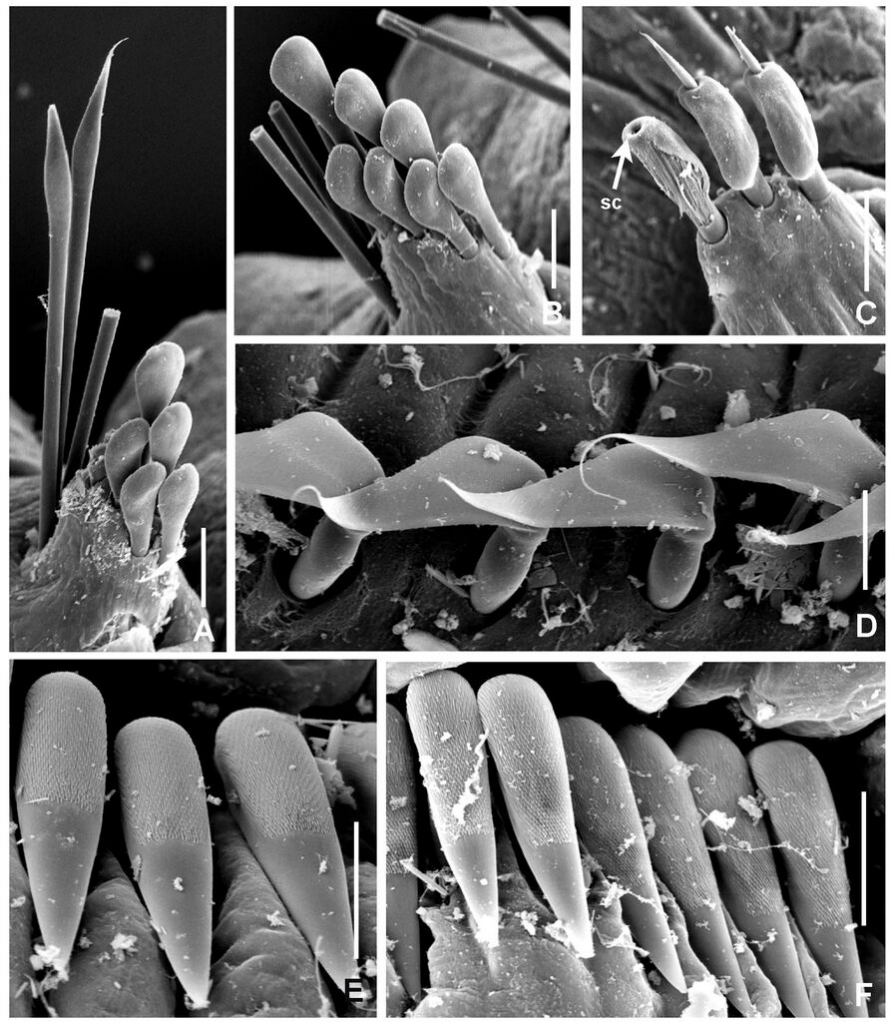
Chaetae of *Anamobaea orstedii* under Scanning Electron Microscopy. A) Thoracic chaetiger: superior group of spinelike chaetae; posterior group with two rows of paleae; B) thoracic paleae, C) abdominal paleae, D) thoracic companion chaetae, E) thoracic uncini, F) abdominal uncini. Abbreviation: sc) scar. Scale bars: A–B) 66.6 μm, C) 50 μm, D–F) 20 μm.

Perkins (1984) also stated that minute mucros were sometimes observed on the concave side of the thoracic palea (to *N. nudicollis* see Perkins 1984 [figure 28E–F]). These were apparently not developed or were broken. In Nishi et al. (2017) [figure 3B–C], the thoracic palea analysed under a Scanning Electron Microscopy (SEM) showed a small scar on the top (not in the concave side of palea), but these went unnoticed or not mentioned. In this study, mucros or scars in the thoracic palea were not detected in Mexican specimens; perhaps, if present, these are only detectable under SEM. In contrast, possibly the length of the mucro of paleae from the anterior half of the abdomen might be useful for distinguishing species, as well as the shape of chaetae from the posterior half of the abdomen.

### Notaulax californica

(Treadwell, 1906)

9C345C42-CF03-56B4-9E33-874BCA435740

Potamilla californica Treadwell, 1906 (Treadwell 1906): 1178.
Hypsicomus sp.— Hartman 1942: 133 (holotype of *P. californica*).
Hypsicomus californicus .— Hartman 1956: 258, 262, 270; Hartman 1969: 701–702 (holotype of *P. californica*).
Notaulax californica .— Perkins 1984: 342–343, fig. 31 (holotype of *P. californica*).-- Yáñez-Rivera et al. 2020: 24–26, Figs. 12A–C.

#### Materials

- **Type status:** Other material. **Occurrence:** catalogNumber: UANL 0335; recordedBy: Jesús Angel de León-González; individualCount: 4; **Taxon:** phylum: Annelida; class: Polychaeta; order: Sabellida Levinsen, 1883; family: Sabellidae Latreille, 1825; genus: Notaulax Tauber, 1879; specificEpithet: *californica* (Treadwell, 1906); **Location:** higherGeographyID: Pacific Ocean; higherGeography: Tropical Eastern Pacific; continent: America; waterBody: Gulf of California; country: México; countryCode: MX; stateProvince: Baja California Sur; municipality: Mulegé; locality: Bahía Concepción, Playa Santispac; verbatimLatitude: 26°45’52.58”N; verbatimLongitude: 111°53’14.88”W; **Identification:** identifiedBy: Jesús Angel de León-González; **Event:** samplingProtocol: By hand; eventDate: May 20, 1985; year: 1985; month: 7; day: 20; habitat: Dead coral; **Record Level:** language: Spanish; institutionID: Universidad Autónoma de Nuevo León; collectionID: Colección Poliquetológica, Universidad Autónoma de Nuevo León; institutionCode: UANL; collectionCode: UANL, NL INV 0002-05-09
- **Type status:** Other material. **Occurrence:** catalogNumber: UANL 0336; recordedBy: Jesús Angel de León-González; individualCount: 14; **Taxon:** phylum: Annelida; class: Polychaeta; order: Sabellida Levinsen, 1883; family: Sabellidae Latreille, 1825; genus: Notaulax Tauber, 1879; specificEpithet: *californica* (Treadwell, 1906); **Location:** higherGeographyID: Pacific Ocean; higherGeography: Tropical Eastern Pacific; continent: America; waterBody: Gulf of California; country: México; countryCode: MX; stateProvince: Baja California Sur; municipality: Mulegé; locality: Bahía Concepción, Playa Requesón; verbatimLatitude: 26°38’15.01”N; verbatimLongitude: 111°49’52.80”W; **Identification:** identifiedBy: Jesús Angel de León-González; **Event:** samplingProtocol: By hand; eventDate: July 20, 1985; year: 1985; month: 7; day: 20; habitat: Dead coral; **Record Level:** language: Spanish; institutionID: Universidad Autónoma de Nuevo León; collectionID: Colección Poliquetológica, Universidad Autónoma de Nuevo León; institutionCode: UANL; collectionCode: UANL, NL INV 0002-05-09
- **Type status:** Other material. **Occurrence:** catalogNumber: UANL 8134; recordedBy: José María Aguilar-Camacho and Irving Daniel Ramírez-Santana; individualCount: 5; **Taxon:** phylum: Annelida; class: Polychaeta; order: Sabellida Levinsen, 1883; family: Sabellidae Latreille, 1825; genus: Notaulax Tauber, 1879; specificEpithet: *californica* (Treadwell, 1906); **Location:** higherGeographyID: Pacific Ocean; higherGeography: Tropical Eastern Pacific; continent: America; waterBody: Gulf of California; country: México; countryCode: MX; stateProvince: Sinaloa; municipality: Ahome; locality: Topolobampo; maximumDepthInMeters: 1 m; verbatimLatitude: 25°33.345’N; verbatimLongitude: 109°05.74’W; **Identification:** identifiedBy: María Ana Tovar-Hernández; **Event:** samplingProtocol: By hand; eventDate: August 9, 2011; year: 2011; month: 8; day: 9; habitat: Buoy fouling; **Record Level:** language: Spanish; institutionID: Universidad Autónoma de Nuevo León; collectionID: Colección Poliquetológica, Universidad Autónoma de Nuevo León; institutionCode: UANL; collectionCode: UANL, NL INV 0002-05-09
- **Type status:** Other material. **Occurrence:** catalogNumber: UANL 8135; recordedBy: José María Aguilar-Camacho and Irving Daniel Ramírez-Santana; individualCount: 8; sex: 4 females, 4 males; lifeStage: Adult; reproductiveCondition: Ripe; **Taxon:** phylum: Annelida; class: Polychaeta; order: Sabellida Levinsen, 1883; family: Sabellidae Latreille, 1825; genus: Notaulax Tauber, 1879; specificEpithet: *californica* (Treadwell, 1906); **Location:** higherGeographyID: Pacific Ocean; higherGeography: Tropical Eastern Pacific; continent: America; waterBody: Gulf of California; country: México; countryCode: MX; stateProvince: Sinaloa; municipality: Ahome; locality: Topolobampo; maximumDepthInMeters: 1 m; verbatimLatitude: 25°34.097’N; verbatimLongitude: 109°04.361’W; **Identification:** identifiedBy: María Ana Tovar-Hernández; **Event:** samplingProtocol: By hand; eventDate: August 9, 2011; year: 2011; month: 8; day: 9; habitat: Buoy fouling; **Record Level:** language: Spanish; institutionID: Universidad Autónoma de Nuevo León; collectionID: Colección Poliquetológica, Universidad Autónoma de Nuevo León; institutionCode: UANL; collectionCode: UANL, NL INV 0002-05-09
- **Type status:** Other material. **Occurrence:** catalogNumber: UANL 8136; recordedBy: María Ana Tovar-Hernández; individualCount: 1; lifeStage: juvenile; **Taxon:** phylum: Annelida; class: Polychaeta; order: Sabellida Levinsen, 1883; family: Sabellidae Latreille, 1825; genus: Notaulax Tauber, 1879; specificEpithet: *californica* (Treadwell, 1906); **Location:** higherGeographyID: Pacific Ocean; higherGeography: Tropical Eastern Pacific; continent: America; waterBody: Gulf of California; country: México; countryCode: MX; stateProvince: Sinaloa; municipality: Mazatlán; locality: Mazatlán, Canal de Navegación; maximumDepthInMeters: 1 m; verbatimLatitude: 23°12’13’’N; verbatimLongitude: 106°24’30.1’’W; **Identification:** identifiedBy: María Ana Tovar-Hernández; **Event:** samplingProtocol: By hand; eventDate: March 24, 2009; year: 2009; month: 3; day: 24; habitat: Buoy fouling; **Record Level:** language: Spanish; institutionID: Universidad Autónoma de Nuevo León; collectionID: Colección Poliquetológica, Universidad Autónoma de Nuevo León; institutionCode: UANL; collectionCode: UANL, NL INV 0002-05-09
- **Type status:** Other material. **Occurrence:** catalogNumber: UANL 8137; recordedBy: Tulio Fabio Villalobos-Guerrero TF and José María Aguilar-Camacho; individualCount: 1; **Taxon:** phylum: Annelida; class: Polychaeta; order: Sabellida Levinsen, 1883; family: Sabellidae Latreille, 1825; genus: Notaulax Tauber, 1879; specificEpithet: *californica* (Treadwell, 1906); **Location:** higherGeographyID: Pacific Ocean; higherGeography: Tropical Eastern Pacific; continent: America; waterBody: Gulf of California; country: México; countryCode: MX; stateProvince: Baja California Sur; municipality: La Paz; locality: La Paz, Club de Yates Palmira; maximumDepthInMeters: 1 m; verbatimLatitude: 24°10.992’N; verbatimLongitude: 110°18.185’W; **Identification:** identifiedBy: María Ana Tovar-Hernández; **Event:** samplingProtocol: By hand; eventDate: August 15, 2011; year: 2011; month: 8; day: 15; habitat: Dock fouling; **Record Level:** language: Spanish; institutionID: Universidad Autónoma de Nuevo León; collectionID: Colección Poliquetológica, Universidad Autónoma de Nuevo León; institutionCode: UANL; collectionCode: UANL, NL INV 0002-05-09

#### Description

**Figure 6.**
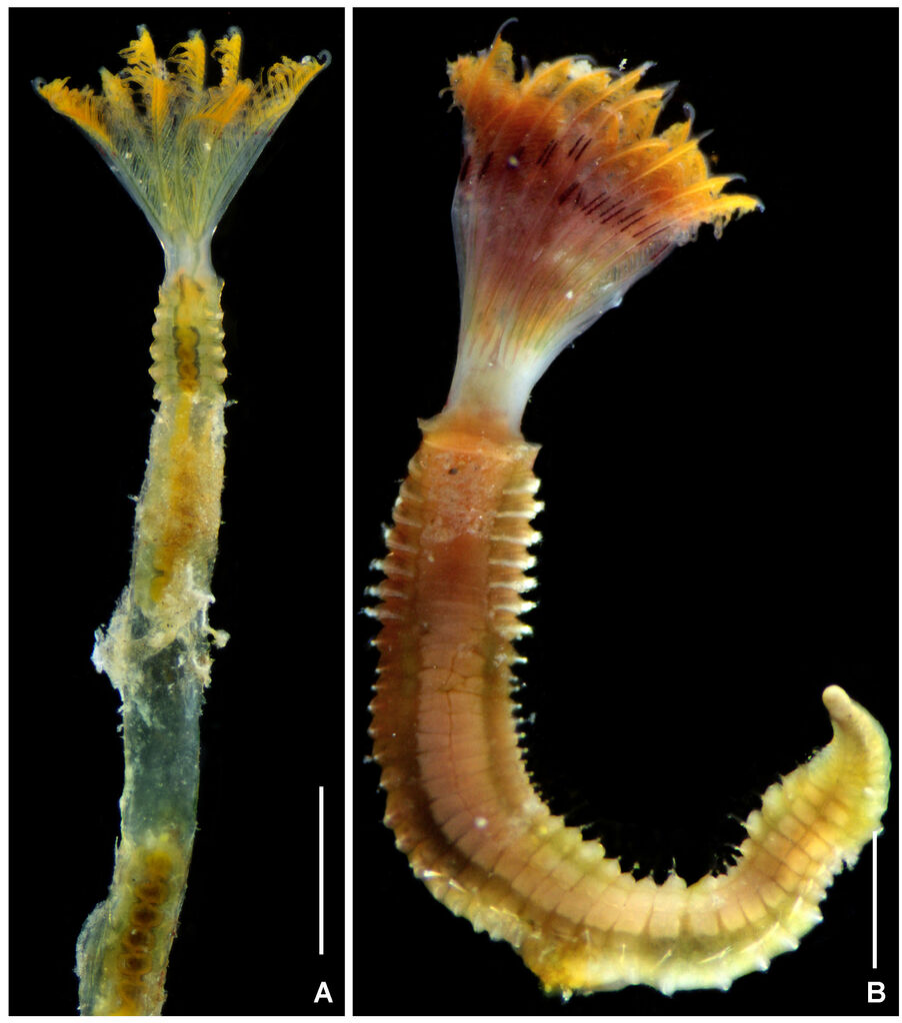
Alive specimens of *Notaulax californica* (Treadwell, 1906). A) Dorsal view, tube partially removed, B) ventral view, tube removed. Scale bars: A–B) 2.6 mm. A–B) UANL 8134.

**Figure 7.**
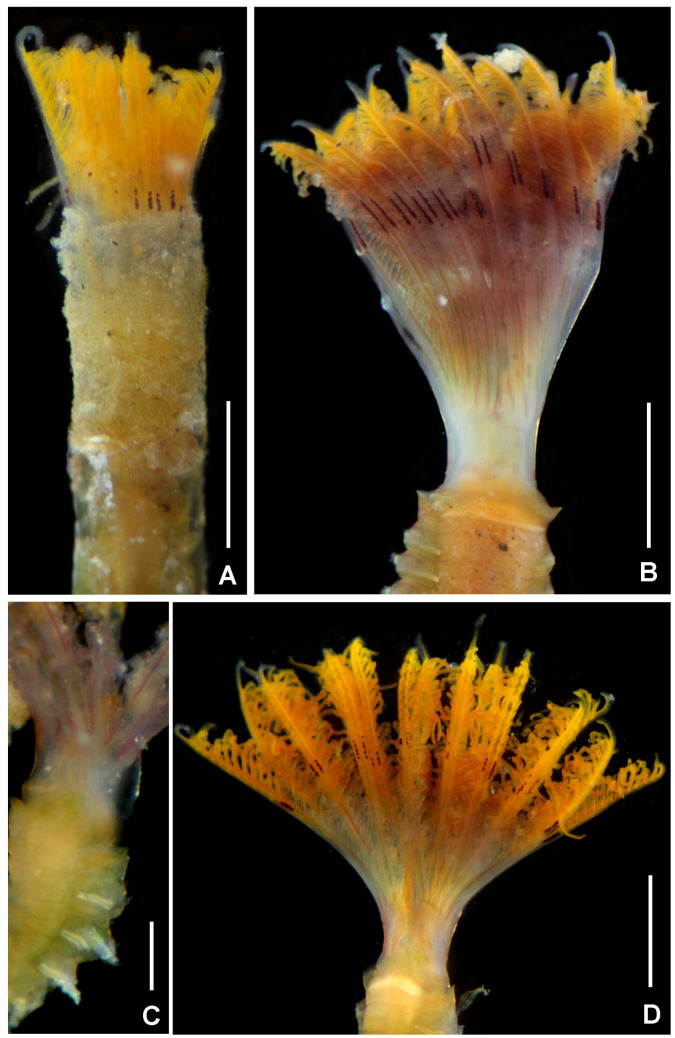
Alive specimens of *Notaulax californica* (Treadwell, 1906). A) Crown partially inside tube, B, D) crown and collar, ventral views, C) thoracic tori, ventral view. Scale bars: A) 1.5 mm, B, D) 1.3 mm, C) 0.3 mm. A–D) UANL 8134.

**Figure 8.**
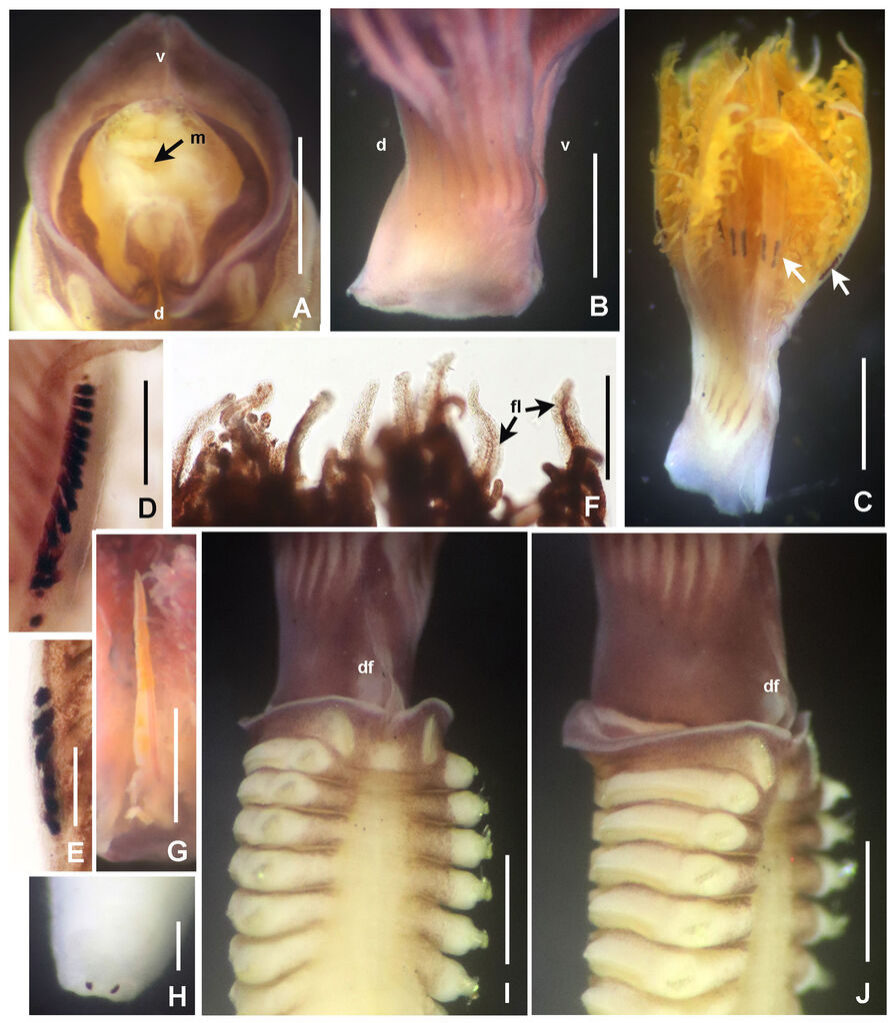
Selected features of *Notaulax californica* (Treadwell, 1906). A) Peristomium, frontal view, crown removed, B) base of radiolar crown, lateral view, C) radiolar crown with arrows pointed to eye bands, D) radiolar ocelli of largest specimen, E) radiolar ocelli of smallest specimen, F) radiolar tips, G) dorsal lip, H) pygidial eyes, I) thorax and base of radiolar crown, dorsal view, J) same, lateral view. Scale bars: A) 0.5 mm, B–C) 1 mm, D–E) 0.15 mm, F, H) 0.2 mm, G) 0.75 mm, I–J) 0.5 mm. Abbreviations: d) dorsal, df) dorsal flange, fl) flanges, m) mouth, v) ventral. A–J) UANL 8135.

**Figure 9.**
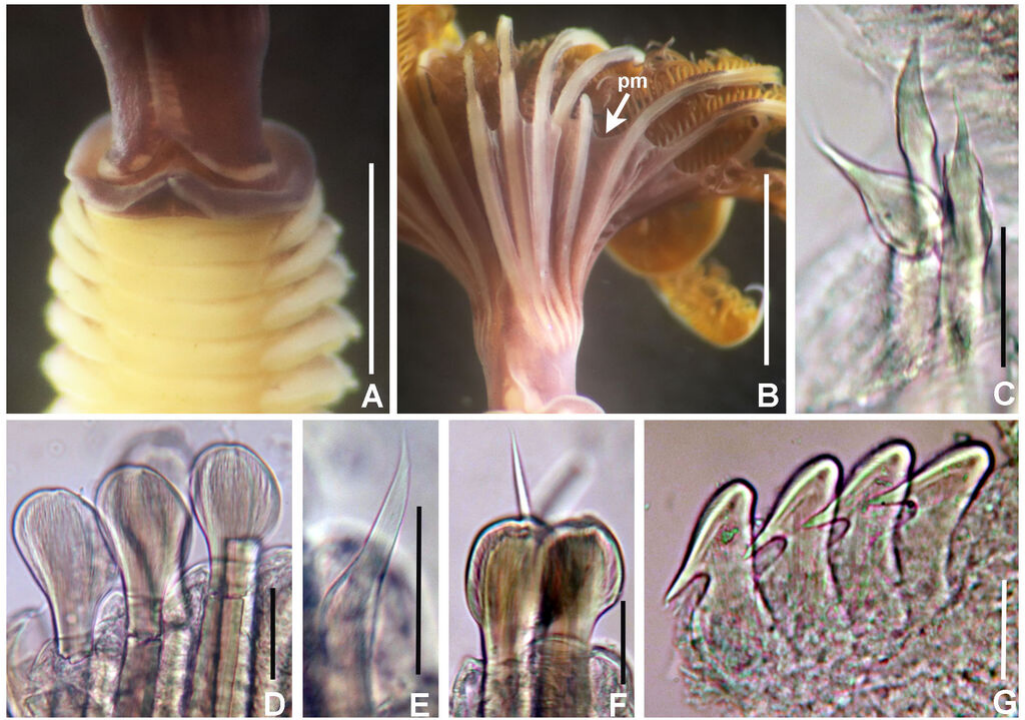
Selected features of *Notaulax californica* (Treadwell, 1906). A) Collar and first thoracic segments, ventral view, B) palmate membrane, C) chaetae from collar, D) paleae from thorax, E) broadly-hooded thoracic chaetae, F) paleae from abdomen, G) abdominal uncini. Scale bars: A) 1.3 mm, B) 2.5 mm, C, E) 40 μm, D, F) 30 μm, G) 24 μm, Abbreviation: pm) palmate membrane. A–I) UANL 8134.

**Figure 10.**
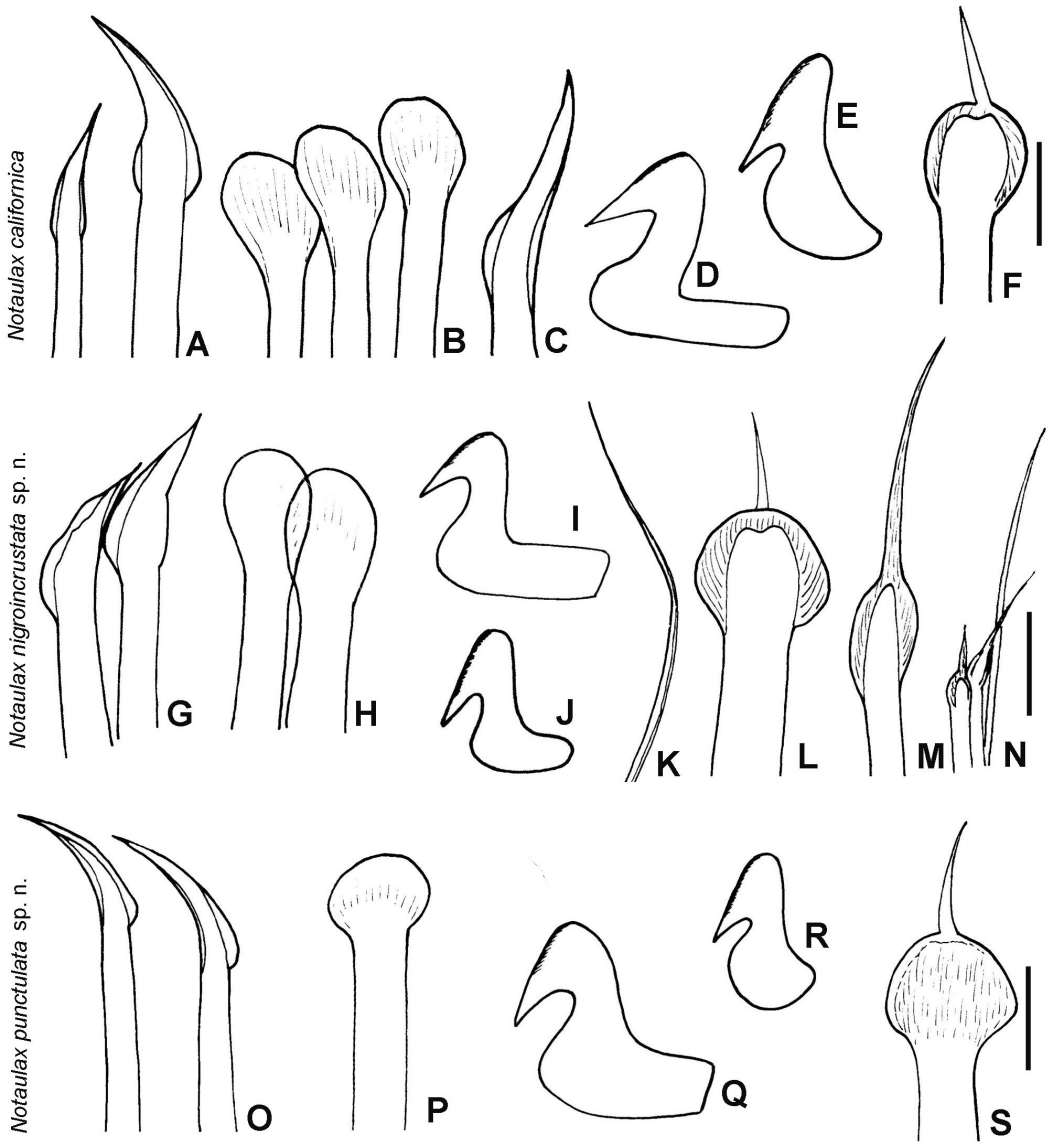
Chaetae and uncini of species in *Notaulax*. A–F) *Notaulax californica* (Treadwell, 1906), UANL 8134; G–N) *N. nigroincrustata* sp. n., UANL 8138 holotype; O–S) *N. punctulata* sp. n., UANL 8143 paratype. A, G, O) chaetae from collar, B, H, P) paleae from thorax, C) broadly-hooded thoracic chaeta, D, I, Q) thoracic uncini, E, J, R) abdominal uncini, F, L, S) paleae from anterior abdomen, K) abdominal, elongate, narrowly-hooded chaeta, M) palea from posterior abdomen, N) posterior abdominal chaetiger. Scale bars: 30 μm.

Figures 6–9 and 10A–F

*Body shape and size*. Specimens flattened dorso-ventrally along the body, ripe specimens with pyriform abdomen in transverse section. Body length 5.2–11.4 mm (X = 8.15 mm, n = 7), width 0.5–1.8 mm (X = 1.02 mm, n = 7).

*Radiolar crown.* Length 1.7–4.8 mm (X = 2.92, n = 7 mm) with 6–11 pairs of radioles (X = 9 pairs of radioles, n = 7). Base of radiolar crown (basal lamina or radiolar lobes) short, as long as the length of first three segments in lateral view (Fig. 8I–J). Dorsal flanges prominent (triangular in side view), ventral flanges reduced (Fig. 8B–C, I–J). Radioles not inrolled mid-ventrally. Radioles united by a palmate membrane as long as 1/2 the length of radiolar crown (Fig. 9B) (or as long as two times the length of base of crown or basal lamina). Radioles not sectioned to count the number of skeletal cells (see remarks to genus level). Longest pinnules located at three quarters of the radiolar crown length. Radiolar tips with broad flanges and short, digitiform tips (Fig. 8G), occupying the space of five pinnules. Radioles with 5–6 black ocelli in single rows on both outer sides of the radioles in smallest specimens (Fig. 8C, E); 14–16 ocelli in largest specimen (Fig. 8D), all bands of ocelli located at 3/4 of the radiolar crown length, each band as long as space of 4–6 pinnules. Dorsal lips as long as a half of the radiolar crown length, orange, erect, with mid-rib (Fig. 8G). Ventral lips short, ear-shaped. Dorsal and ventral pinnular appendages absent.

*Peristomium.* Anterior peristomial ring not exposed beyond collar (not visible), high, rounded, slightly longer ventrally. Posterior peristomial ring collar: dorsal collar margins fused to faecal groove (Fig. 8I). Lateral collar margins entire (Fig. 8J). Ventral collar margins incised, forming two shallow, rounded lappets (Fig. 9A). Ventral sacs and parallel lamellae absent.

*Thorax.* Chaetiger 1: with straight oblique rows of spine-like notochaetae (Fig. 8I–J). Spine-like chaetae from collar with variable shape: stouter, straight with blades terminated by an elongated conical hood inflated at the base, usually bent or wavy in the slender distal half; and slender, sharply curved at the end, provided on the convex side with a short obliquely-striated wing (Fig. 9C and Fig. 10A). Ventral shield rectangular, whitish (Fig. 7B and D). Chaetigers 2–8: notopodia with superior groups of short, spine-like chaetae (Fig. 9E and Fig. 10C) and inferior groups of paleate chaetae without mucros (Fig. 9D and Fig. 10B). Neurochaetae avicular uncini, handles as long as two times the length of crest, developed breast and several rows of minute, similarly-sized teeth occupying half of crest (Fig. 9D and Fig. 10D); neuropodial companion chaetae with rounded denticulate head and long, gently tapering asymmetrical membrane. Ventral shields broad, rectangular, laterally indented by neuropodial tori (Fig. 7B–C and Fig. 9A).Fig. 10

*Abdomen.* Segments: 64–76 chaetigers in complete specimens (n = 2). Abdominal ventral shields dark brown, rectangular, divided longitudinally by faecal groove. Anterior abdominal segments with paleate notochaetae, nearly rounded with mucros as long as palea width (Fig. 9F and Fig. 10F) and elongate, narrowly-hooded chaetae. Avicular uncini similar to thoracic ones, but handles short (handle as long as the length of crest) and dentition covering 3/4 of the crest (Fig. 9G and Fig. 10E). Posterior abdominal segments with oval paleae with a mucro longer than three times the palea width and modified, elongate narrowly-hooded chaetae. Pygidium rounded with two black, large, reniform eyespots (Fig. 8H).

*Variation.* The number of radiolar ocelli may change according to ontogeny. Smallest specimens from the same lot have only 5–6 ocelli per row, whereas largest specimens which are ripe have 14–16 ocelli per row.

*Colour in live specimens*. Body yellow-greenish with ventral shields cream-coloured (Fig. 6A–B). Radiolar crown with basal half translucent-whitish (Fig. 7B and D); distal half orange with rows of black ocelli located at three quarters of radiolar crown length (Fig. 6B, Fig. 7A–B and D).

*Colour in preserved specimens*. Body yellow (Fig. 8I–J) or pale (Fig. 8H), peristomium purple (Fig. 9A) and collar and some areas of thoracic segments purple (Fig. 8I–J). Basal half of radiolar crown purple or whitish (Fig. 7B–C).

*Tubes*: Organic tubes, covered with fine sand anteriorly near mouth, translucent posteriorly (Fig. 6A).

#### Diagnosis

Ventral margin of collar incised, forming rounded lappets. Short bands of radiolar ocelli (each band as long as the space of 4–6 pinnules), ocelli distributed in single rows of five ocelli (in smallest, juvenile specimen) to 16 ocelli ocelli (largest, ripe specimens), bands located at three quarters of the radiolar crown length (Table 2).

#### Biology

Females with oocytes in anterior abdomen and males with spermatozoa along abdomen (UANL 8135) with spherical nucleus and rounded cap-like acrosome.

#### Taxon discussion

*Potamilla californica* was described from Monterey Bay, California by Treadwell (1906). It was included in Hartman (1942) as *Hypsicomus* sp. and later as *Hypsicomus californica* (Hartman 1969). The original description is very brief and does not includes figures (Treadwell 1906). In the description provided by Hartman (1969), it is mentioned that the collar is incised mid-ventrally and she emphasises the similarity of *Hypsicomus lyra* Moore in Moore and Bush (originally described from Suruga Bay, Japan) and *Hypsicomus californica*, except for the absence of radiolar ocelli in the second species. Later, Perkins (1984) had the opportunity to review and re-describe the holotype housed at the National Museum of Natural History (USNM 5222), transferring it to the genus *Notaulax* and confirming the presence of radiolar ocelli.

Perkins (1984) also suggest that *N. californica* is not the same as *N. lyra* as suggested by Hartman (1969), mainly based of chaetal differences. However, to judge the original description of *N. lyra*, there is another difference between both species: *N. lyra* may present 5–20 ocelli exhibiting much irregularity in arrangement and seldom occupy the entire distance in individual radioles, ocelli may be widely separated, much crowded or even coalesced. *Notaulax californica* has a low and consistent number of ocelli per radiole (5–6), these are always located at 3/4 of the radiolar length and close together one to another.

Fouling specimens here reviewed from the Gulf of California match with descriptions of the holotype provided by Hartman (1969) and Perkins (1984) to *N. californica*, as well as those records by *Yáñez-Rivera et al. (2020)* to Bahía de Chamela Islands Sanctuary, Jalisco (Mexico). Some specimens are embedded within their tubes and are hard to remove from these in order to count abdominal segments.

Data available for species of *Notaulax* suggest the presence of gonochorism, with gametes distributed in the abdominal segments, sperm developing in tetrads and having spherical heads: *Notaulax midoculi* (Hoagland, 1919), *N. nudicollis*, *N. occidentalis* (Baird, 1865) (Rouse and Fitzhugh 1994), *N. lyra* (Moore and Bush, 1904) (Sanders 2014) and *N. tilosaula* (Schmarda, 1861) (Tovar-Hernández et al. 2017). This is confirmed here to *Notaulax californica*, as well as to the two new species described below.

### Notaulax nigroincrustata

Tovar-Hernández, García-Garza & de León-González sp. n.

6857B8C3-59F0-516E-829E-975944EF7B03

D4F1D931-2B60-4CF9-BC16-ABEA71ADAD43

#### Materials

- **Type status:** Holotype. **Occurrence:** catalogNumber: UANL 8138; recordedBy: José María Aguilar-Camacho and Irving Daniel Ramírez-Santana; sex: male; lifeStage: adult; reproductiveCondition: ripe; **Taxon:** phylum: Annelida; class: Polychaeta; order: Sabellida Levinsen, 1883; family: Sabellidae Latreille, 1825; genus: Notaulax Tauber, 1879; **Location:** higherGeographyID: Pacific Ocean; higherGeography: Tropical Eastern Pacific; continent: America; waterBody: Gulf of California; country: México; countryCode: MX; stateProvince: Baja California Sur; municipality: La Paz; locality: La Paz, Marina La Paz; maximumDepthInMeters: 1 m; verbatimLatitude: 24° 09.296’ N; verbatimLongitude: 110° 19.566’ W; **Identification:** identifiedBy: María Ana Tovar-Hernández; **Event:** samplingProtocol: By hand; eventDate: August 14, 2011; year: 2011; month: 8; day: 14; habitat: Dock fouling; **Record Level:** language: Spanish; institutionID: Universidad Autónoma de Nuevo León; collectionID: Colección Poliquetológica, Universidad Autónoma de Nuevo León; institutionCode: UANL; collectionCode: UANL, NL INV 0002-05-09
- **Type status:** Paratype. **Occurrence:** catalogNumber: UANL 8139; recordedBy: José María Aguilar-Camacho and Irving Daniel Ramírez-Santana; individualCount: 2; sex: male; lifeStage: adult; reproductiveCondition: ripe; **Taxon:** phylum: Annelida; class: Polychaeta; order: Sabellida Levinsen, 1883; family: Sabellidae Latreille, 1825; genus: Notaulax Tauber, 1879; **Location:** higherGeographyID: Pacific Ocean; higherGeography: Tropical Eastern Pacific; continent: America; waterBody: Gulf of California; country: México; countryCode: MX; stateProvince: Baja California Sur; municipality: La Paz; locality: La Paz, Marina La Paz; maximumDepthInMeters: 1 m; verbatimLatitude: 24° 09.296’ N; verbatimLongitude: 110° 19.566’ W; **Identification:** identifiedBy: María Ana Tovar-Hernández; **Event:** samplingProtocol: By hand; eventDate: August 14, 2011; year: 2011; month: 8; day: 14; habitat: Dock fouling; **Record Level:** language: Spanish; institutionID: Universidad Autónoma de Nuevo León; collectionID: Colección Poliquetológica, Universidad Autónoma de Nuevo León; institutionCode: UANL; collectionCode: UANL, NL INV 0002-05-09
- **Type status:** Paratype. **Occurrence:** catalogNumber: UANL 8140; recordedBy: Tulio Fabio Villalobos-Guerrero and José María Aguilar-Camacho; individualCount: 3; sex: 1 female, 2 males; lifeStage: adult; reproductiveCondition: ripe; **Taxon:** phylum: Annelida; class: Polychaeta; order: Sabellida Levinsen, 1883; family: Sabellidae Latreille, 1825; genus: Notaulax Tauber, 1879; **Location:** higherGeographyID: Pacific Ocean; higherGeography: Tropical Eastern Pacific; continent: America; waterBody: Gulf of California; country: México; countryCode: MX; stateProvince: Baja California Sur; municipality: La Paz; locality: La Paz, Marina La Paz; maximumDepthInMeters: 1 m; verbatimLatitude: 24° 09.318’ N; verbatimLongitude: 110° 19.630’ W; **Identification:** identifiedBy: María Ana Tovar-Hernández; **Event:** samplingProtocol: By hand; eventDate: August 14, 2011; year: 2011; month: 8; day: 14; habitat: Dock fouling; **Record Level:** language: Spanish; institutionID: Universidad Autónoma de Nuevo León; collectionID: Colección Poliquetológica, Universidad Autónoma de Nuevo León; institutionCode: UANL; collectionCode: UANL, NL INV 0002-05-09
- **Type status:** Other material. **Occurrence:** catalogNumber: UANL 8141; recordedBy: José María Aguilar-Camacho and Irving Daniel Ramírez-Santana; individualCount: 14; **Taxon:** phylum: Annelida; class: Polychaeta; order: Sabellida Levinsen, 1883; family: Sabellidae Latreille, 1825; genus: Notaulax Tauber, 1879; **Location:** higherGeographyID: Pacific Ocean; higherGeography: Tropical Eastern Pacific; continent: America; waterBody: Gulf of California; country: México; countryCode: MX; stateProvince: Baja California Sur; municipality: La Paz; locality: La Paz, Canal de Navegación; maximumDepthInMeters: 1 m; verbatimLatitude: 24° 16.447’ N; verbatimLongitude: 110° 19.852’ W,; **Identification:** identifiedBy: María Ana Tovar-Hernández; **Event:** samplingProtocol: By hand; eventDate: August 14, 2011; year: 2011; month: 8; day: 1; habitat: Hull fouling; **Record Level:** language: Spanish; institutionID: Universidad Autónoma de Nuevo León; collectionID: Colección Poliquetológica, Universidad Autónoma de Nuevo León; institutionCode: UANL; collectionCode: UANL, NL INV 0002-05-09

#### Description

**Figure 11.**
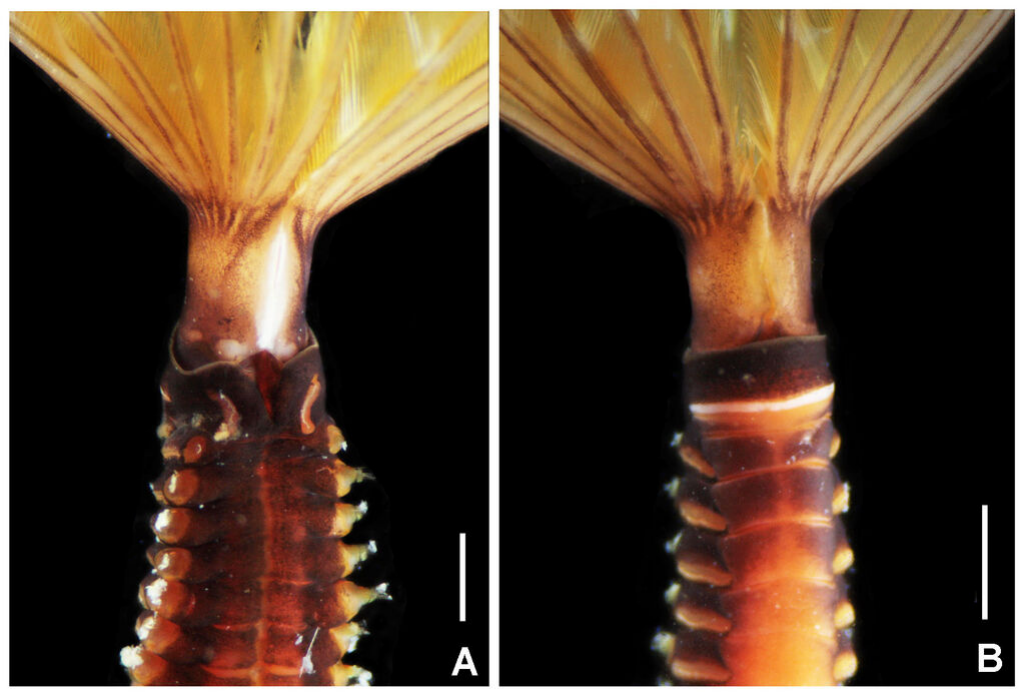
Alive holotype of *Notaulax nigroincrustata* sp. n. A) Base of crown and thorax, dorsal view, B) same, ventral view. Scale bars: A) 1 mm, B) 2 mm. A–B) UANL 8138 holotype.

**Figure 12.**
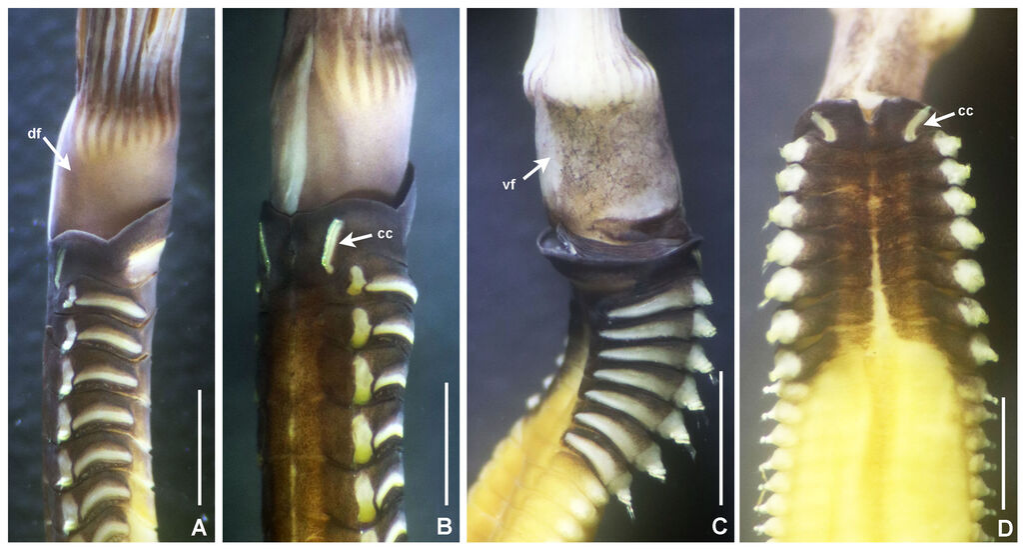
Selected features of *Notaulax nigroincrustata* sp. n. A) Base of crown and thorax, lateral view, B) same, dorso-lateral view, C) same, ventro-lateral view, D) thorax, dorsal view. Scale bars: A–B) 1 mm, C) 1.5 mm, D) 2 mm, Abbreviations: cc) collar chaetiger, df) dorsal flange, vf) ventral flange. A–F) UANL 8139 paratypes.

**Figure 13.**
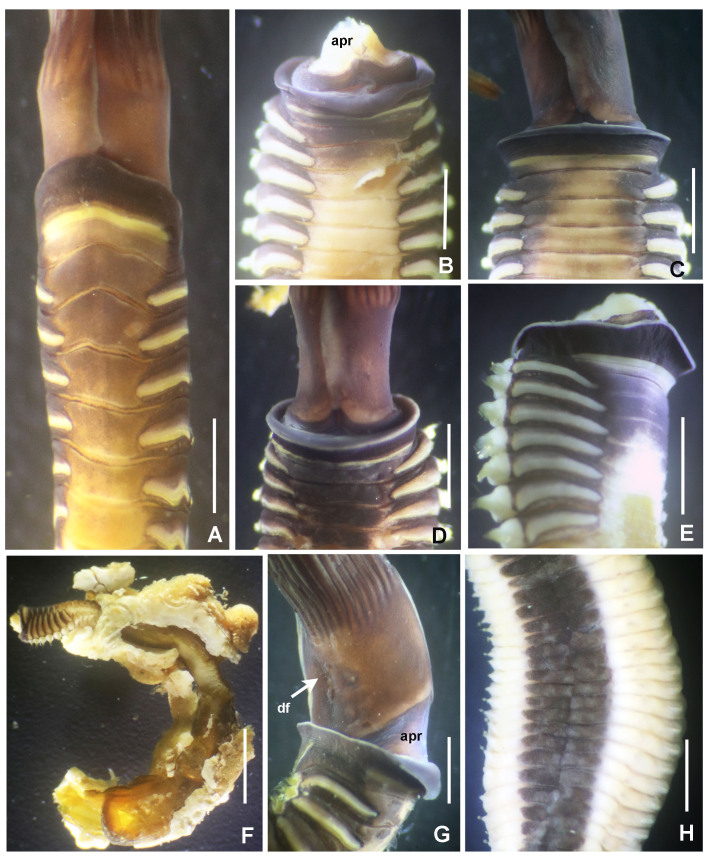
Selected features of *Notaulax nigroincrustata* sp. n. A–E) Collar, different shapes, ventral view, F) sclerezoan worm inside a calcium carbonate matrix, G) base of radiolar crown and peristomium, lateral view, H) abdominal shields. Scale bars: A) 0.65 mm, B) 1 mm, C–D) 1 mm, E) 1.3 mm, F) 2.2 mm, G) 0.75 mm, H) 1 mm. Abbreviations: apr) anterior peristomial ring, df) dorsal flange. A) UANL 8138 holotype, B–H) UANL 8140 paratypes.

**Figure 14.**
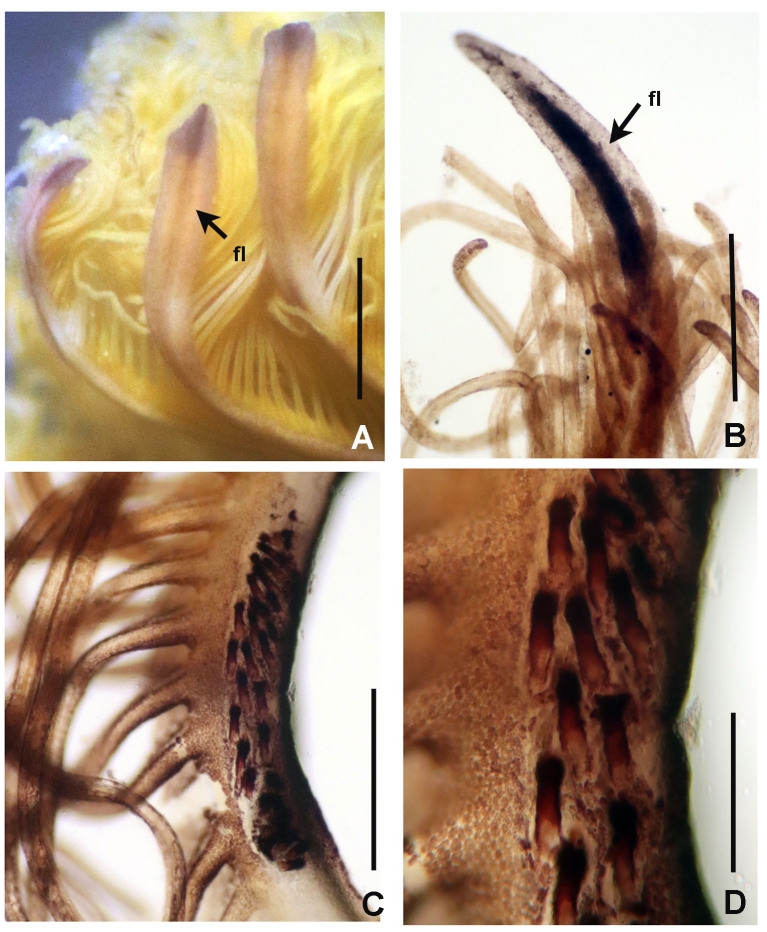
Selected features of *Notaulax nigroincrustata* sp. n. A–B) radiolar tips showing radiolar flanges, C) radiolar ocelli, D) same, detail. Scale bars: A–B) 0.3 mm, C) 0.25 mm, D) 0.1 mm. Abbreviations: fl) flange. A–D) UANL 8138 holotype.

**Figure 15.**
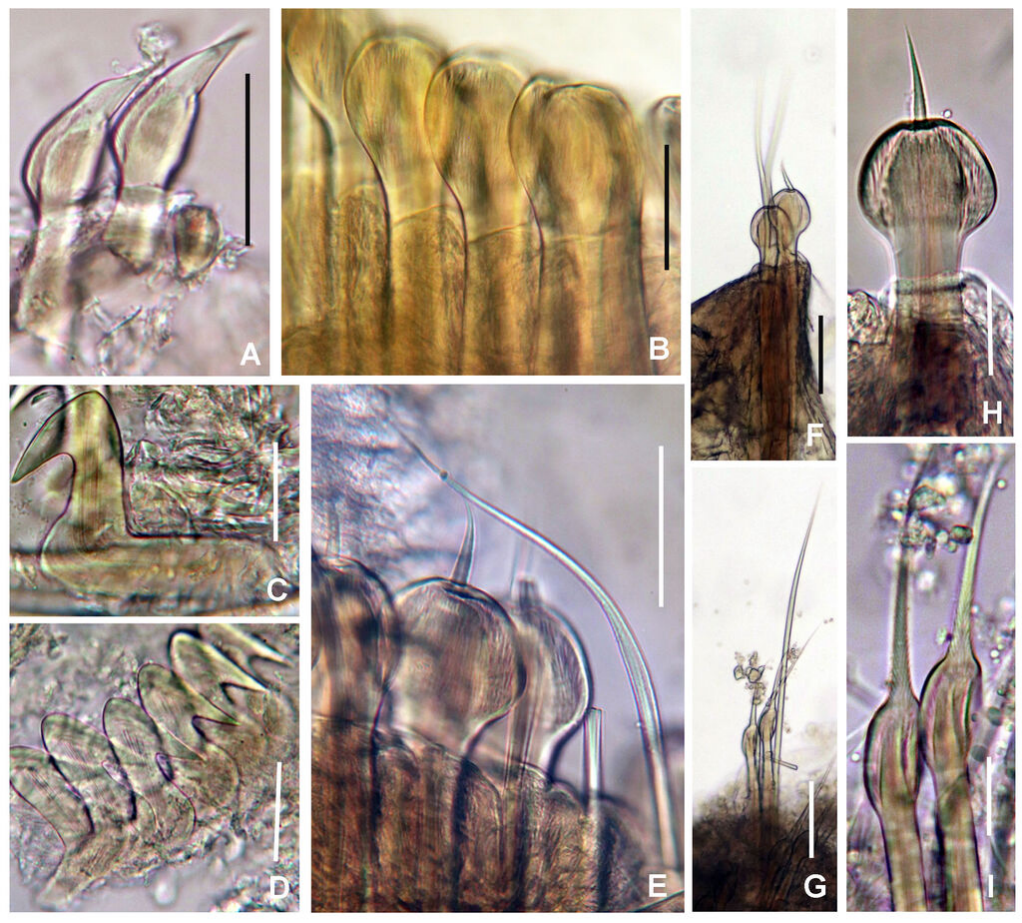
Chaetae of *Notaulax nigroincrustata* sp. n. A) collar chaetae, B) paleae from thorax, C) thoracic uncinus, D) abdominal uncini, E) abdominal paleae and elongate, narrowly-hooded chaeta from anterior abdomen, F) chaetiger from anterior abdomen, G) chaetiger from posterior abdomen, H) palea from anterior abdomen, I) palea from posterior abdomen. Scale bars: A) 40 μm, B, E–I) 30 μm, C–D) 24 μm. A–I) UANL 8138 holotype.

Figures 10G–N and 11–15

*Body shape and trunk size*. Specimens flattened dorso-ventrally along the body, ripe specimens with pyriform abdomen in transverse section. Body length 17.4 mm (6.3–34 mm, X = 14.3 mm, n = 4 paratypes incomplete, lacking some areas of abdomen), 2 mm width (1.2–2.7 mm, X = 2.7 mm, n = 5).

*Radiolar crown.* Length 9.4 mm (8–18 mm, X = 12.66, n = 5 mm) with 12 pairs of radioles (8–15 pairs of radioles, X = 12.8 radioles, n = 5). Radiolar lobes fused dorsally, whose union form a bridge internally (H-shaped). Base of radiolar crown (basal lamina or radiolar lobes) short, as long as the length of five segments in lateral view (Fig. 12) (3,3,3,3,5 segments each paratype). Dorsal flanges prominent (Fig. 11A and Fig. 12A), ventral flanges reduced (Fig. 11B and Fig. 13C). Radioles not inrolled mid-ventrally. Radioles fused by a palmate membrane, about 1/2 length of radiole length. There are thirteen pinnules above the palmate membrane and a group of 26–30 black ocelli in an oval group on both outer sides of the radioles, each group as long as the space of eight pinnules (Fig. 14C–D). Radiolar flanges broad (Fig. 14A–B). Radioles not sectioned to count the number of skeletal cells (see remarks to genus level). Longest pinnules located at three quarters of the radiolar crown length. Radiolar tips flattened, erect, with broad flanges and digitiform tips, black or purple coloured (Fig. 14B), occupying the space of 10 pinnules. Dorsal lips as long as 1/4 of the radiolar crown length, orange, erect, with mid-rib. Ventral lips short, ear-shaped. Dorsal and ventral pinnular appendages absent.

*Peristomium.* Anterior peristomial ring not exposed beyond collar (not visible), high, dome-shaped (Fig. 13B). Posterior peristomial ring collar: dorsal collar margins fused to faecal groove (Fig. 12D). Lateral collar margins entire (Fig. 12A, C and Fig. 13G) or with an asymmetrical lateral notch V-shaped (Fig. 12A). Ventral collar margins entire, slightly higher than lateral collar margin (Fig. 11B and Fig. 13A–E). Ventral lappets, ventral sacs and parallel lamellae absent.

*Thorax.* Chaetiger 1: with straight oblique rows of spine-like notochaetae (Fig. 10G and Fig. 12A–B) or slightly curved basally (Fig. 11A and Fig. 12D). Spine-like chaetae from collar with variable shape: stouter, straight with blades terminated by an elongated conical hood inflated at the base, usually bent or wavy in the slender distal half; and slender, sharply curved at the end (Fig. 10G and Fig. 15A). Ventral shield rectangular, with an anterior whitish transverse band (Fig. 11B and Fig. 13A), sometimes covered partially by anterior margin of ventral collar (Fig. 13B). Chaetigers 2–8: notopodia with superior group of short, spine-like chaetae and inferior groups of paleate chaetae without mucros. Neurochaetae avicular uncini, with handles as long as two times the length of crest, developed breast (Fig. 10I and Fig. 15C) and several rows of minute, similarly-sized teeth occupying half of crest length (Fig. 15C); neuropodial companion chaetae with rounded denticulate head and long, gently tapering asymmetrical membrane. Ventral shields broad, trapezoidal, laterally indented by neuropodial tori (Fig. 13A–E).

*Abdomen.* A total of 142 abdominal chaetigers (9-64 chaetigers, X = 24.8; n = 5, all incomplete specimens). Abdominal ventral shields dark brown, rectangular, divided longitudinally by faecal groove (Fig. 13H). Anterior abdominal segments with paleate notochaetae, nearly rounded with mucros as long as palea width (Fig. 10L, Fig. 15I, andJ–K) and elongate, narrowly-hooded chaetae (Fig. 10K, Fig. 15E, F and H). Avicular abdominal uncini similar to thoracic ones but handles shorter (as long as the length of crest) and dentition covering 3/4 of the crest length (Fig. 10J and Fig. 15D). Posterior abdominal segments with oval paleae with a mucro longer than three times the palea width (Fig. 10M, Fig. 15G and I) and modified, elongate narrowly-hooded chaetae (Fig. 10N and Fig. 15G). Pygidium unknown (incomplete specimens)Fig. 10

*Variation*: Amongst the set of specimens here reviewed, two were found with the lateral collar margin V-shaped, apparently natural, not damaged. Perkins (1984) (p. 338) attributed this variation to ontogeny in specimens grouped within “*N. nudicollis*”: collar of single lobe on adults or 2- or 4-lobed on juveniles. However, both specimens from La Paz with the lateral collar margin V-shaped were one of the largest (+34 mm) and one of medium-size (+13 mm). Thus, this anomaly cannot be attributed to ontogeny. In addition, the length of the basal lamina is also variable amongst specimens: it is as long as the length of three to five segments in lateral view (3,3,3,3,4,5,5, n = 7).

*Colour in live specimens*. Black thorax dorsally (Fig. 11A). Collar ventral black with a white broad, transverse band near the base of the collar (Fig. 11B). Anterior three thoracic segments black on ventral side (Fig. 11B). Segments 4–8 with black colour surrounding each torus and ventral shields orange-cream coloured (Fig. 11B). Base of radiolar crown purple-whitish (Fig. 11A–B). Radiolar rachis and radiolar tips purple. Pinnules orange alternating with white and purple bands (Fig. 11B). Ventral abdominal shields brown or black, with lateral sides of body yellow or cream-coloured.

*Colour in preserved specimens* (eight years post-fixation): black colour in life turns to deep purple-black in post-fixed specimens (Fig. 12A–D). Other areas are cream-coloured (Fig. 12C–D). Ventral abdominal shields purple (Fig. 13H). Radiolar rachis and radiolar tips purple (Fig. 14A–B).

*Tubes*: Organic, horny tube, translucent, some bioclaustrated in a carbonated matrix (sclerozoan) (Fig. 13F).

#### Diagnosis

Ventral margin of collar entire. Short bands of radiolar ocelli (each band as long as the space of seven pinnules), ocelli distributed in oval groups of 26–30 ocelli each, groups located at the middle of the radiolar crown length.

#### Etymology

The name is a compound adjective, where the first term refers to the black colour of the worm (Latin *nigrum*, meaning black) and the second adjective (Latin *incrustatĭo*, meaning encrustation) makes reference to the encrusting nature of the species on the docks and hulls of the ships where the species was found.

#### Ecology

In La Paz (Gulf of California), *Notaulax nigroincrustata* sp. n, was found in hull fouling in densities of 16–40 ind.m^-2^. Additionally, it is remarkable that the black body pigmentation remains up tonine years after sampling.

#### Biology

Holotype (UANL 8138) and paratypes (UANL 8139, 8140) males with a huge mass of sperm in abdomen. Ripe worms have abdomen pyriform in cross section with a dorsal hump full of sperm. Males from additional samples (UANL 8141) have sperm between the body wall and internal layer of tubes. Spermatozoa has a spherical nucleus and rounded cap-like acrosome. Paratype female (UANL 8140) has full-developed oocytes in abdomen.

#### Taxon discussion

In his revision of *Notaulax*, Perkins (1984) includes specimens from Florida, Virgin Islands, North Carolina, Puerto Rico, Brazil, West Africa and western Mexico under the name of *Notaulax nudicollis* Krøyer, arguing minor differences amongst them attributed to geographic separation. However, as stated below, specimens from western Mexico, analysed in this study, have peculiar features that allow us the recognition of a separated species.

Specimens here reviewed from the Gulf of California, match only with the specimens reported from Zihuatanejo (western Mexico) by Perkins (1984) as “*N. nudicollis*”. Specimens from Zihuatanejo and adult specimens from La Paz have groups of up to about 30 ocelli, separated from palmate membrane by shorter length; and a collar entire ventrally. The holotype of *Notaulax nudicollis* has tight, elongate-oval groups of 30–70 ocelli, diminishing in number distally to form a row and separated from palmate membrane by about the length of the membrane; minute mucros in thoracic palea and mucro from the anterior abdominal palea are shorter than the palea width Perkins (1984). In specimens from western Mexico (Gulf of California, La Paz), ocelli are distributed in oval groups of 26–30 ocelli, not forming a row distally and that group is located 13 pinnules above the palmate membrane (less than a half of palmate membrane length); minute or reminiscent mucros in thoracic palea are not present; and mucros of paleate chaetae from the anterior abdomen are as long as the palea width. As these are features were constant in examined specimens, we decided to establish a new name for that species.

Amongst other species of *Notaulax* distributed in Western Mexico, *N. nigroincrustata* sp. n., differs from *N. californica* by the presence of an entire ventral collar margin (incised forming ventral lappets in *N. californica*); and radiolar ocelli distributed in groups (single rows in *N. californica*) (Table 2). *Notaulax nigroincrustata* sp. n. differs from *N. punctulata* sp. n. by the absence of interramal eyespots (present in *N. punctulata* sp. n.), amongst other features (Table 2).

### Notaulax punctulata

Tovar-Hernández, García-Garza & de León-González sp. n.

82DF61FD-29A9-5227-A329-CC2C18422EC7

42F8AC0E-C4D8-4894-B046-EC4A73C6A31A

#### Materials

- **Type status:** Holotype. **Occurrence:** catalogNumber: UANL 8142; recordedBy: Tulio Fabio Villalobos-Guerrero; sex: Female; lifeStage: Adult; reproductiveCondition: Ripe; **Taxon:** phylum: Annelida; class: Polychaeta; order: Sabellida Levinsen, 1883; family: Sabellidae Latreille, 1825; genus: Notaulax Tauber, 1879; **Location:** higherGeographyID: Pacific Ocean; higherGeography: Tropical Eastern Pacific; continent: America; country: México; countryCode: MX; stateProvince: Guerrero; municipality: Acapulco de Juárez; locality: Acapulco, Playa Hornitos; maximumDepthInMeters: 3 m; verbatimLatitude: 16°51’26.41”N; verbatimLongitude: 99°53’20.70”W; **Identification:** identifiedBy: María Ana Tovar-Hernández; **Event:** samplingProtocol: By hand; eventDate: November 9, 2015; year: 2015; month: 11; day: 9; habitat: Sclerozoan of oyster attached to dock; **Record Level:** language: Spanish; institutionID: Universidad Autónoma de Nuevo León; collectionID: Colección Poliquetológica, Universidad Autónoma de Nuevo León; institutionCode: UANL; collectionCode: UANL, NL INV 0002-05-09
- **Type status:** Paratype. **Occurrence:** catalogNumber: UANL 8143; recordedBy: Tulio Fabio Villalobos-Guerrero; individualCount: 1; sex: Male; lifeStage: Adult; reproductiveCondition: Ripe; **Taxon:** phylum: Annelida; class: Polychaeta; order: Sabellida; family: Sabellidae; genus: Notaulax; **Location:** higherGeographyID: Pacific Ocean; higherGeography: Tropical Eastern Pacific; continent: America; country: México; countryCode: MX; stateProvince: Guerrero; municipality: Acapulco de Juárez; locality: Acapulco, Playa Hornitos; maximumDepthInMeters: 3 m; verbatimLatitude: 16°51’26.41”N; verbatimLongitude: 99°53’20.70”W; **Identification:** identifiedBy: María Ana Tovar-Hernández; **Event:** samplingProtocol: By hand; eventDate: November 9, 2015; year: 2015; month: 11; day: 9; habitat: Sclerozoan of oyster attached to dock; **Record Level:** language: Spanish; institutionID: Universidad Autónoma de Nuevo León; collectionID: Colección Poliquetológica, Universidad Autónoma de Nuevo León; institutionCode: UANL; collectionCode: UANL, NL INV 0002-05-09

#### Description

**Figure 16.**
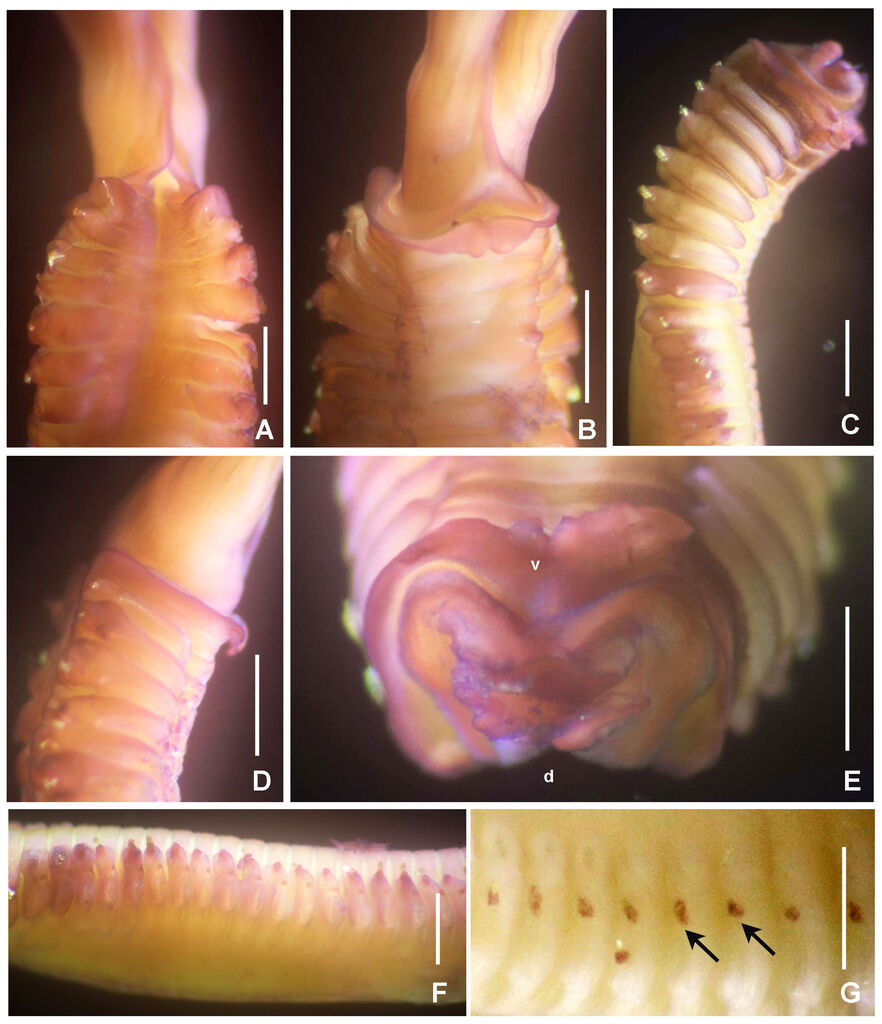
Selected features of *Notaulax punctulata* sp. n. A) Base of crown and thorax, dorsal view, B) same, ventral view, C) thorax and anterior abdomen, lateral view, D) collar, lateral view, E) peristomium, frontal view, crown removed, F–G) interramal eyespots on abdomen as pointed with arrows. A–B, D–F) stained with Shirla stain A. Scale bars: A–G) 0.5 mm. Abbreviations: d) dorsal, v) ventral. A–B, D, F–G) UANL 8142 holotype, C, E) UANL 8143 paratype.

**Figure 17.**
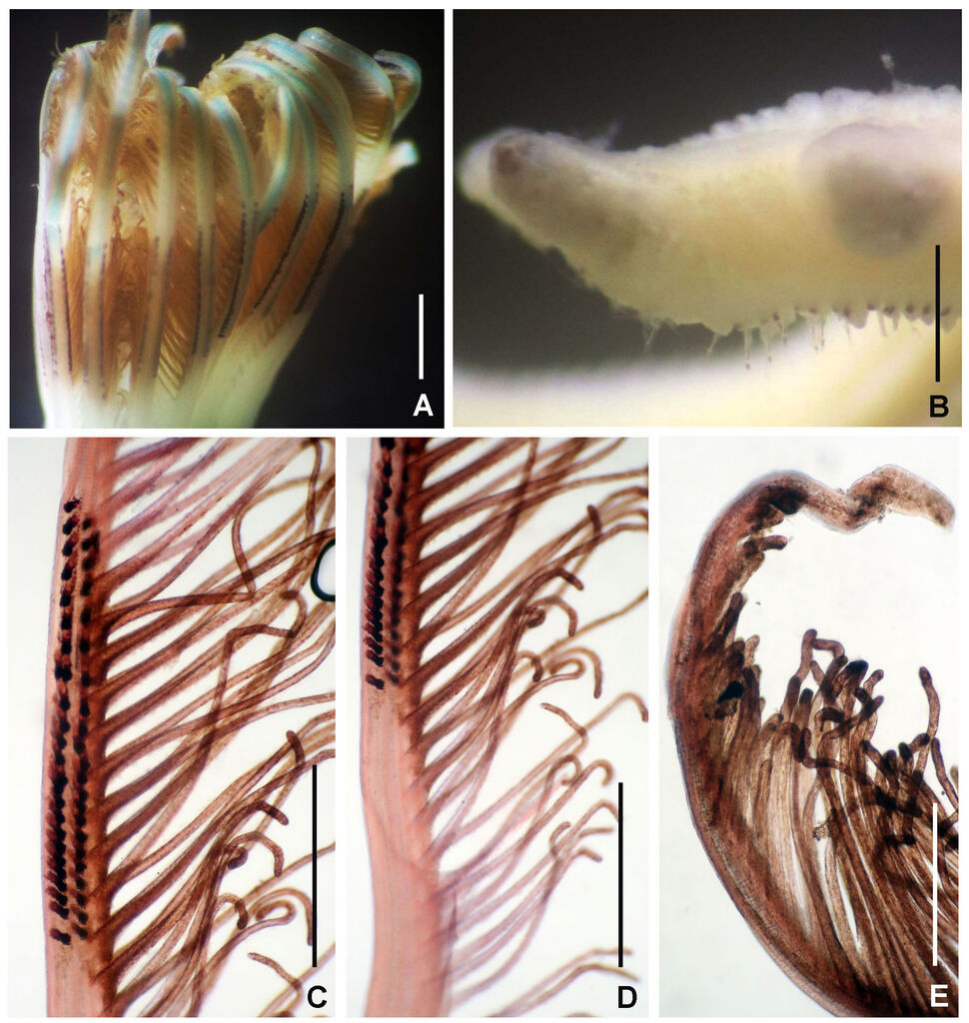
Selected features of *Notaulax punctulata* sp. n. A) Anterior part of crown, B) pygidium and interramal eyespots, C) radiolar bands of ocelli, D) palmate membrane, E) radiolar tip. Scale bars: A–E) 0.4 mm. A–D) UANL 8142 holotype.

**Figure 18.**
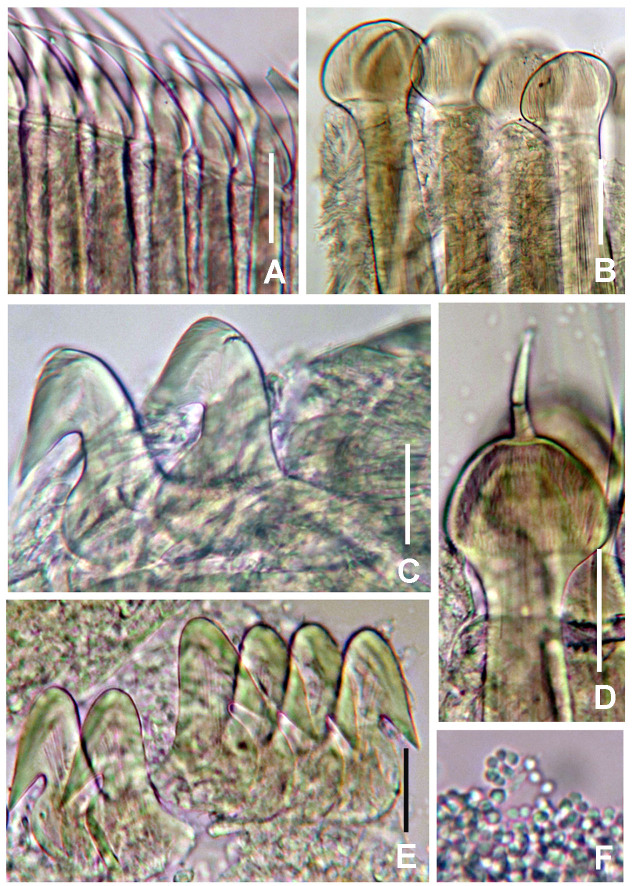
Chaetae of *Notaulax punctulata* sp. n. A) Chaetae from collar, B) paleae from thorax, C) thoracic uncini, D) palea from abdomen, E) abdominal uncini, F) sperm. Scale bars: A) 40 μm, B, D) 30 μm, C, E) 24 μm, F) 1000x magnification. A–F) UANL 8143 paratype.

Figures 10O–S, 16–18

*Body shape and trunk size*. Specimens flattened dorso-ventrally along the thorax, with pyriform abdomen in transversal section. Body length 15.4 mm (+13,2 mm), 1.5 mm (1.8 mm width).

*Radiolar crown.* Length 4.9 mm with 11 pairs of radioles. Radiolar lobes fused dorsally. Base of radiolar crown (basal lamina) short, as long as the length of three segments in lateral view, with dorsal and ventral flanges reduced (Fig. 16A–B and D). Radioles fused by a palmate membrane, about 1/2 of radiole length. Immediately above the membrane, there is a group of 24–26 black ocelli in a single row on both outer sides of the radioles, each band as long as the space of 13 pinnules (Fig. 17A and C–D). Radiolar flanges broad (Fig. 17A). Radiolar tips flattened, with broad flanges and long digitiform tips, occupying the space of 12 pinnules (Fig. 17E). Longest pinnules located at three quarters of the radiolar crown length. Radioles not sectioned to count the number of skeletal cells (see remarks to genus level). Dorsal lips as long as 1/4 of the radiolar crown length, erect, with mid-rib. Ventral lips short, ear-shaped. Dorsal and ventral pinnular appendages absent.

*Peristomium.* Anterior peristomial ring not exposed beyond collar (not visible). Posterior peristomial ring collar: dorsal collar margins fused to faecal groove (Fig. 16A). Lateral collar margins entire (Fig. 16D). Ventral collar margins incised forming rounded lappets (Fig. 16B, E). Ventral sacs and parallel lamellae absent.

*Thorax.* Chaetiger 1: with straight oblique rows of spine-like notochaetae (Fig. 16A). Spine-like chaetae from collar short, stouter, straight with blades sharply curved at the end (Fig. 10O and Fig. 18A). Ventral shield rectangular. Chaetigers 2–8: notopodia with superior group of short, spine-like chaetae and inferior groups of paleate chaetae without mucros (Fig. 10P and Fig. 18B). Neurochaetae avicular uncini, with handles as long as two times the length of crest, developed breast and several rows of minute, similarly-sized teeth occupying half of the crest (Fig. 10Q, andFig. 18C); neuropodial companion chaetae with rounded denticulate head and long, gently tapering asymmetrical membrane. Ventral shields broad, trapezoidal, laterally indented by neuropodial tori.

*Abdomen.* Segments 142 (83 abdominal segments). Interramal eyespots present (Fig. 16F–G and Fig. 17B). Abdominal ventral shields brown, rectangular, divided longitudinally by faecal groove. Anterior abdominal segments with paleate notochaetae, nearly rounded with mucros as long as palea width (Fig. 10S and Fig. 18B) and elongate, narrowly-hooded chaetae. Avicular abdominal uncini similar to thoracic ones, but handles shorter (as long as the crest length) and dentition covering 3/4 of the crest (Fig. 10R and Fig. 18F). Posterior abdominal segments with oval paleae with a mucro longer than three times the palea width and modified, elongate narrowly-hooded chaetae. Pygidium with two rounded black eyes.

*Colour in live specimens*: Unknown.

*Colour in preserved specimens*: Body pale with black interramal eyespots (Fig. 16G, andFig. 17B); collar and some areas of thorax purple (Fig. 16C).

*Tubes*: Organic, horny, translucent tubes.

#### Diagnosis

Ventral margin of collar incised, forming rounded lappets. Long bands or radiolar ocelli (each band as long as the space of 13 pinnules), ocelli distributed in single rows of 24 ocelli each, bands located at the middle of the radiolar crown length. Interramal eyespots on abdominal segments.

#### Etymology

The specific epithet *punctulata* is the feminine of the Latin word *punctulatus*, meaning 'having small spots or punctures' or 'punctulate' and refers to the presence of abdominal interramal eyespots of the species.

#### Biology

Holotype female with fully-developed oocytes in abdomen. Paratype male with sperm in abdomen, spermatozoa with spherical nucleus and rounded cap-like acrosome (Fig. 18F).

#### Taxon discussion

Abdominal interramal eyespots have been only reported in *Notaulax tilosaula* (Schmarda, 1861) (Schmarda 1861) by Tovar-Hernández et al. (2017). *Notaulax punctulata* sp. n. differs from *N. tilosaula* by the following features: the palmate membrane extends until a half of the radiolar crown length in *N. punctulata* sp. n. (1/4 of the radiolar crown length in *N. tilosaula*); radiolar tips are very long, occupying space of 10–12 pinnules width in *N. punctulata* sp. n. (short tips in *N. tilosaula*, occupying space of three pinnules width; band of ocelli in *N. punctulata* sp. n. occupying space of 13 pinnules (25 pinnules in *N. tilosaula*); ventral lappets rounded in *N. punctulata* sp. n. (triangular in *N. tilosaula*); lateral collar margin level in *N. punctulata* sp. n. (V-shaped margin in *N. tilosaula*) and mucro of posterior abdominal paleae as long as three times the width of paleae (mucro as long as five times the width of paleae in *N. tilosaula*).

Amongst the species of *Notaulax* from the Mexican Pacific, *N. punctulata* sp. n. is unique by the presence of interramal eyespots on the abdominal segments (Table 2). *Notaulax californica* and *N. punctulata* sp. n. have radiolar ocelli distributed in single rows, but in the former, there are a minor number (5–6), whereas in the second, there may be up to 24 ocelli per row. *Notaulax nigroincrustata* sp. n. have ocelli distributed in oval groups of 26–30 ocelli each.

## Discussion

This study provides some new morphological characters and reproductive issues for the species of *Anamobaea* and *Notaulax* as a first step towards a better understanding of their anatomy and reproduction with some potential applications to their phylogeny. However, the requirement should be emphasised for a worldwide revision to *Notaulax*, because descriptions of many species are brief; type material of some species are lost; *Notaulax phaeotaenia* (Schmarda) has been widely reported, but their “cosmopolitanism” requires corroboration, amongst other cases. As shown in this contribution, some features used before in descriptions, such as number of radiolar ocelli, is growth-dependent; consequently, revision of different developmental stages are needed.

Four species of *Notaulax* have been found fouling in docks, buoys and hulls of ships: *N. tilosaula* (Tovar-Hernández et al. 2017), *N. californica*, *N. nigroincrustata* sp. n. and *N. punctulata* sp. n. (present study). These species could be used in monitoring programmes for the detection of invasive species in marinas and ports. Additionally, the estimated densities of *N. nigroincrustata* sp. n. as a fouling material in the Gulf of California (16–40 ind.m^-2^) will allow further studies on reproduction, ontogeny and ecology.

## Supplementary Material

XML Treatment for Anamobaea

XML Treatment for Anamobaea orstedii

XML Treatment for Notaulax

XML Treatment for Notaulax californica

XML Treatment for Notaulax nigroincrustata

XML Treatment for Notaulax punctulata

## Figures and Tables

**Table 1.** Comparison of the genera *Anamobaea* Krøyer, 1856, *Hypsicomus* Grube, 1870 and *Notaulax* Tauber, 1879.

**Feature**	*** Anamobaea ***	*** Hypsicomus ***	*** Notaulax ***
Palmate membrane	Present	Present	Present
Radiolar flanges	Absent	Present	Present
Radiolar ocelli	Present	Present	Present
Dorsal pinnular appendages	Present	Absent	Absent
Dorsal lips with radiolar appendages	Present	Present	Present
Ventral lips	Present	Present	Present
Paralel lamellae	Present	Present	Present
Ventral sacs	Present	Present	Present
Peristomial eyes	Absent	?	Absent
Anterior margin of anterior peristomial ring	High, triangular, ventrally longer (present study) *versus* low, of even height all around (Fitzhugh 1989)	Low, of even height all around (Fitzhugh 1989)	Low, of even height all around (Fitzhugh 1989, Capa et al. 2019) *versus* high, rounded, slightly longer ventrally (Tovar-Hernández et al. 2020, present study)
Flanges on base of radiolar crown	Present, dorsal and ventrally, erect, prominent	Absent	Present, dorsal and ventrally, less developed than *Anamobaea*
Accessory lamellae	Absent	Present, dorsal pair curved, rounded distally; ventral pair collar-like	Absent
Arrangement of chaetae in collar fascicle	In a bunch	In a bunch	Longitudinal, oblique, L-shaped, J-shaped, C-shaped
Number of thoracic chaetigers	20 to 73 (usually near 50)	9–13 (*H. stichophthalmos* (Grube 1863)	Usually 8, but Capa and Murray (2015) reported 8–26 in *N.* sp. 3
Thoracic tori	Contacting ventral shields	Not contacting ventral shields	Contacting ventral shields
Mucro of thoracic paleate chaetae	Absent	Absent or present	Absent or reminiscent
Abdominal chaetae	*Anterior abdomen*: modified, elongate, narrowly hooded; and paleate with short mucros (as long as paleae width) *Posterior abdomen*: modified, elongate, narrowly-hooded chaetae; and palea with long mucros (longer than three times the palea width) and spherical palea	*Anterior abdomen*: elongate, narrowly hooded; and paleate with short mucros (as long as paleae width) *Posterior abdomen*: modified, elongate, narrowly hooded chaetae; ? not described	*Anterior abdomen*: elongate, narrowly hooded; and paleate with short mucros (as long as paleae width) *Posterior abdomen*: modified, elongate, narrowly-hooded chaetae; and paleate chaetae with long mucros (longer than three times the paleae width) and spherical or oval paleae
Reproduction	Simultaneous hermaphroditism	?	Gonochorism

**Table 2.** Comparison of *Notaulax* species from the Mexican Pacific.

Species	*Notaulax californica* Treadwell, 1906	*Notaulax nigroincrustata* sp. n.	*Notaulax punctulata* sp. n.
Record or type locality	Topolobampo, Sinaloa; Bahía Concepción, Baja California Sur, Gulf of California (Record)	La Paz, Baja California Sur, Gulf of California (type locality)	Acapulco, Guerrero, western Mexico (type locality)
Ventral margin of collar	Incised	Entire	Incised
Lateral margin of collar	Even in height	Even in height or rarely V-shaped	Even in height
Radiolar ocelli	5–6 ocelli in single row in smallest specimens, 14–15 ocelli in largest specimen	26–30 ocelli in oval group	24 ocelli in single rows
Length of bands or groups of ocelli	As long as space of 4–6 pinnules	As long as space of eight pinnules	As long as space of 13 pinnules
Base of radiolar crown (basa lamina)	Short (as long as three segments)	Short to medium length (as short as 3, 4 or 5 thoracic segments)	Short (as long as three thoracic segments)
Interramal eyespots on abdominal segments	Absent	Absent	Present
Location of ocelli	3/4 of the radiolar crown length	1/2 of the radiolar crown length	1/2 of the radiolar crown length
Extension of palmate membrane	Basal half of the radiolar crown length (1/2)	Basal half of the radiolar crown length (1/2)	Basal half of the radiolar crown length (1/2)
Chaetiger 1	Straight, oblique	Straight, oblique or slightly curved basally	Straight, oblique
Abdominal paleae	Anterior abdominal segments: spherical with short mucros. Posterior abdominal segments: oval with long mucros.	Anterior abdominal segments: spherical with short mucros. Posterior abdominal segments: oval with long mucros.	Anterior abdominal segments: spherical with short mucros. Posterior abdominal segments: oval with long mucros.
